# Potentiating CD8^+^ T cell antitumor activity by inhibiting PCSK9 to promote LDLR-mediated TCR recycling and signaling

**DOI:** 10.1007/s13238-021-00821-2

**Published:** 2021-02-19

**Authors:** Juanjuan Yuan, Ting Cai, Xiaojun Zheng, Yangzi Ren, Jingwen Qi, Xiaofei Lu, Huihui Chen, Huizhen Lin, Zijie Chen, Mengnan Liu, Shangwen He, Qijun Chen, Siyang Feng, Yingjun Wu, Zhenhai Zhang, Yanqing Ding, Wei Yang

**Affiliations:** 1 Shunde Hospital, Southern Medical University (The First People’s Hospital of Shunde), Foshan, 528308 China; 2 Guangdong Provincial Key Laboratory of Molecular Oncologic Pathology, Department of Pathology, School of Basic Medical Sciences, Southern Medical University, Guangzhou, 510515 China; 3 Department of Pathology, Nanfang Hospital, Southern Medical University, Guangzhou, 510515 China; 4 Guangdong Provincial Key Laboratory of Molecular Oncologic Pathology, Southern Medical University, Guangzhou, 510515 China; 5 Center for Precision Medicine, Guangdong Provincial People’s Hospital, School of Medicine, South China University of Technology, Guangzhou, 510030 China

**Keywords:** LDLR, PCSK9, TCR, CD8^+^ T cells, tumor microenvironment, cancer immunotherapy

## Abstract

Metabolic regulation has been proven to play a critical role in T cell antitumor immunity. However, cholesterol metabolism as a key component of this regulation remains largely unexplored. Herein, we found that the low-density lipoprotein receptor (LDLR), which has been previously identified as a transporter for cholesterol, plays a pivotal role in regulating CD8^+^ T cell antitumor activity. Besides the involvement of cholesterol uptake which is mediated by LDLR in T cell priming and clonal expansion, we also found a non-canonical function of LDLR in CD8^+^ T cells: LDLR interacts with the T-cell receptor (TCR) complex and regulates TCR recycling and signaling, thus facilitating the effector function of cytotoxic T-lymphocytes (CTLs). Furthermore, we found that the tumor microenvironment (TME) downregulates CD8^+^ T cell LDLR level and TCR signaling via tumor cell-derived proprotein convertase subtilisin/kexin type 9 (PCSK9) which binds to LDLR and prevents the recycling of LDLR and TCR to the plasma membrane thus inhibits the effector function of CTLs. Moreover, genetic deletion or pharmacological inhibition of PCSK9 in tumor cells can enhance the antitumor activity of CD8^+^ T cells by alleviating the suppressive effect on CD8^+^ T cells and consequently inhibit tumor progression. While previously established as a hypercholesterolemia target, this study highlights PCSK9/LDLR as a potential target for cancer immunotherapy as well.

**Supplementary Information:**

The online version of this article (10.1007/s13238-021-00821-2) contains supplementary material, which is available to authorized users.

## Introduction

As the killer cells to cancer, CD8^+^ T cells play a central role in cancer immune surveillance. CD8^+^ T cell-based immunotherapy has emerged as one of the most prominent cancer therapeutic strategies. Particular success has been seen with new approaches like immune checkpoint blockade (ICB), targeting PD-1 and CTLA4, and chimeric antigen receptor T (CAR-T) cell therapy, both of which have been approved for the treatment of a variety of cancers (Ishida et al., 1992; Leach et al., 1996; Morgan et al., 2006; Wolchok et al., 2013; Maude et al., 2014). Despite the clinical successes, the efficacy of CD8^+^ T cell-based immunotherapy varies substantially across malignancies and individuals (Rizvi et al., 2015; Neelapu et al., 2018; Rafiq et al., 2020). As such, further investigation into the regulatory mechanisms and efficacy factors of immune therapy is necessary.

Upon antigen stimulation, peripheral CD8^+^ T cells will traffic to the tumor microenvironment (TME) and mediate antitumor immunity (Fu and Jiang, 2018; Hu et al., 2018). However, the TME possesses numerous immunosuppressive properties, primarily mediated by immune suppressive stromal cells, myeloid cells, lymphoid cells and tumor cells themselves, limiting the antitumor activity of CD8^+^ T cells. While these immunosuppressive cells are the main cause of immunotherapy failure (Draghiciu et al., 2015; Kalluri, 2016; Kumar et al., 2017; Mantovani et al., 2017; Togashi et al., 2019), a lack of nutrients—such as glucose and amino acids, as well as hypoxia in the TME are also correlated with CD8^+^ T cell dysfunction (Chang et al., 2015; Bunse et al., 2018; Leone et al., 2019; Baumann et al., 2020; Bian et al., 2020). Furthermore, cellular metabolic regulation has been shown to be critical for T cell differentiation and effector function (Almeida et al., 2016; Kishton et al., 2017; Patel and Powell, 2017). These previous studies suggest that the metabolic regulation by the TME plays a vital role in CD8^+^ T cell suppression and tumor immune evasion (Sukumar et al., 2013; Ho et al., 2015; Zhang et al., 2017; Bian et al., 2020; Wang and Zou, 2020).

As the primary component of metabolic regulation, cholesterol metabolism in particular is essential for CD8^+^ T cell activation, clonal expansion and effector function (Kidani et al., 2013; Wang et al., 2016; Yang et al., 2016). Recent studies have also highlighted the importance of cellular cholesterol metabolism in regulating the antitumor efficacy of CD8^+^ T cells (Yang et al., 2016; Ma et al., 2020). However, the mechanisms by which the TME reprograms CD8^+^ T cell cholesterol metabolism, and to what extent do they impact tumor immune evasion, remain unknown.

To investigate that how cholesterol metabolism modulates CD8^+^ T cell function in the TME, we systematically evaluated cholesterol metabolism in tumor infiltrating CD8^+^ T cells. The results showed that the TME reprogrammed cholesterol metabolism of CD8^+^ T cells, in particular the cholesterol uptake which is mediated by low-density lipoprotein receptor (LDL receptor, LDLR) was dramatically decreased in intratumoral CD8^+^ T cells. Furthermore, we demonstrated that LDLR was essential for CD8^+^ T cell immune response and antitumor immunity. In addition to mediating the uptake of cholesterol, LDLR also interacts with CD3 subunits of the T-cell receptor (TCR) complex, thus modulating TCR recycling and signaling. Moreover, it has been previously reported that proprotein convertase subtilisin/kexin type 9 (PCSK9) regulates the degradation of LDLR, consequently blocking cholesterol uptake (Maxwell et al., 2005; Kwon et al., 2008; Poirier et al., 2008; He et al., 2020). Upon investigation, we found that PCSK9 was highly expressed in tumors, and the tumor cells derived PCSK9 dampened the immune response of CD8^+^ T cells via downregulating LDLR level and ultimately hindered TCR signaling and effector function. These findings highlight the PCSK9-LDLR-TCR regulatory network as a novel potential target in cancer immunotherapy.

## Results

### LDLR deficiency hinders the antitumor activity of CD8^+^ T cells

Antigen stimulation induces cholesterol metabolic reprogramming in CD8^+^ T cells, which enables the cells to acquire sufficient cholesterol to support clonal expansion and effector function (Zech et al., 2009; Kidani et al., 2013; Yang et al., 2016; Newton et al., 2018). The TME has been demonstrated as a hypoxia and nutrient restricted environment (Chang et al., 2015; Zhang and Ertl, 2016; Cascone et al., 2018). Whether there is sufficient cholesterol in the TME to support the antitumor activity of CD8^+^ T cells, and how CD8^+^ T cells acquire sufficient cholesterol in such environment, are little known. To answer these questions, we first analyzed the apolipoprotein B (APOB) level of clinical cancer samples and syngeneic mouse tumor samples. We found that the APOB level, which represents the LDL/cholesterol level, was significantly higher in the tumor regions than that in the paracancerous normal regions (Fig. S1A–F). In contrast, the cellular cholesterol level of tumor infiltrating CD8^+^ T cells from MC38 tumor burdened syngeneic mice was lower than that of the splenic CD8^+^ T cells, when quantified by Filipin III imaging (Fig. S1G and S1H). These findings indicate that the reduced cellular cholesterol in CD8^+^ T cells may be due to the internal alterations of tumor infiltrating CD8^+^ T cells.

**Figure 1:**
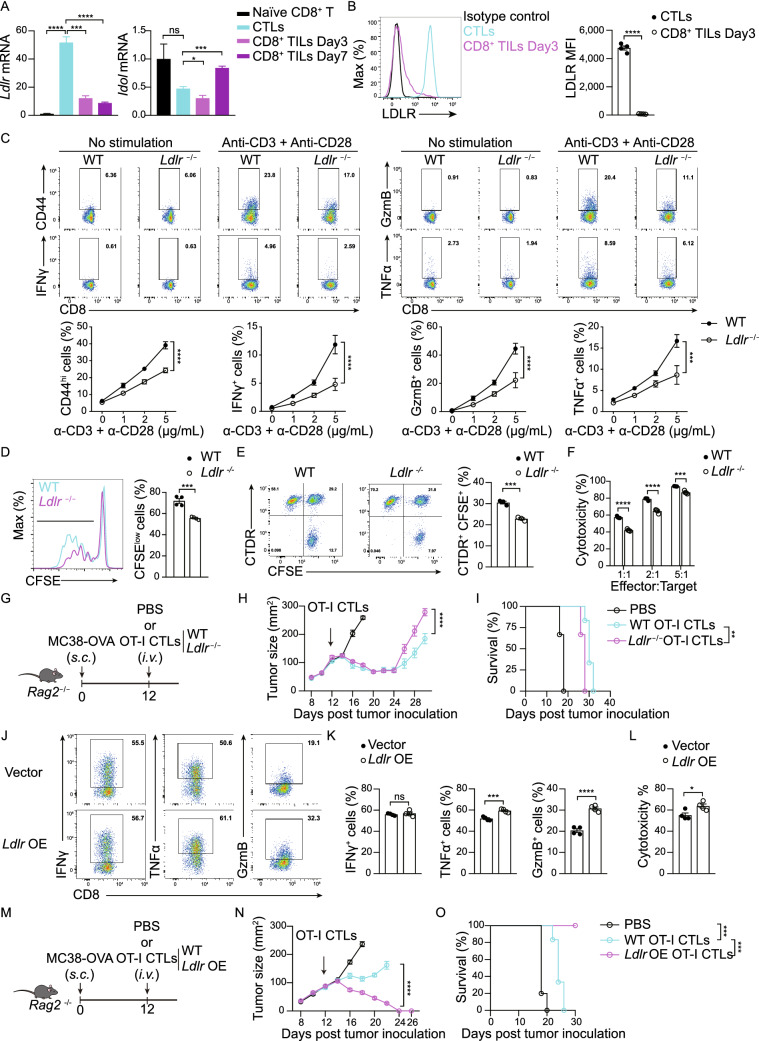
**LDLR deficiency hinders the antitumor activity of CD8**^**+**^**T cells**. (A) Transcriptional level of genes involved in cholesterol transport in naïve CD8^+^ T cells, CTLs and CD8^+^ TILs (isolated at Day3 or Day7 post CTLs adoptive transfer), (*n* = 4). (B) LDLR expression level on CTLs and CD8^+^ TILs (isolated at Day3 post CTLs adoptive transfer), (*n* = 4). (C) Activation and cytokine/granule productions of WT and *Ldlr*^−/−^ CD8^+^ T cells. Naïve CD8^+^ T cells were isolated from the spleen and stimulated with anti-CD3 and anti-CD28 antibodies for 24 h at indicated concentrations. Data were analyzed by two-way ANOVA (*n* = 4). (D) CD8^+^ T cell proliferation was measured by CFSE dilution assay. CD8^+^ T cells were isolated from the spleen and stimulated with 1μg/mL plate-coated anti-CD3 and anti-CD28 antibodies for 72 h, (*n* = 4). (E) Immunological synapse formation of WT and *Ldlr*^−/−^ CTLs. CFSE-labeled CTLs and CellTracker Deep Red (CTDR)-labeled OVA-pulsed EL4 cells were cocultured for 30 min, (*n* = 3). (F) Cytotoxicity of WT and *Ldlr*^−/−^ CTLs. Splenocytes from WT and *Ldlr*^−/−^ OT-I mice were stimulated with OVA_257–264_ and IL-2 to generate mature CTLs. CTLs were incubated with OVA-pulsed CTDR-labeled EL4 cells and CFSE-labeled non-pulsed EL4 cells for 4 h. The ratio of OVA-pulsed and non-pulsed EL4 cells was calculated to determine the cytotoxicity of CTLs, (*n* = 4). (G) Illustration of adoptive transfer of PBS, WT or *Ldlr*^−/−^ CTLs to MC38-OVA tumor-bearing *Rag2*^−/−^ mice. (H and I) Tumor growth (H) and survival (I) of MC38-OVA tumor-bearing *Rag2*^−/−^ mice with CTL transfer as shown in (G), (*n* = 6). (J and K) Cytokine and granule productions of control and *Ldlr* OE CTLs. *Ldlr* was overexpressed in CTLs with retrovirus infection. The sorted cells were stimulated with 1μg/mL plate-coated anti-CD3 and anti-CD28 antibodies for 4 h, (*n* = 4). (L) Cytotoxicity of control and *Ldlr* OE CTLs. CTLs were incubated with OVA-pulsed EL4 cells and non-pulsed EL4 cells for 4 h, (*n* = 4). (M) Illustration of adoptive transfer of PBS, WT or *Ldlr* OE CTLs to MC38-OVA tumor-bearing *Rag2*^−/−^ mice. (N and O) Tumor growth (N) and survival (O) of MC38-OVA tumor-bearing *Rag2*^−/−^ mice with CTL transfer as shown in (M), (*n* = 5–6). Data were analyzed by *t* test (A, B, D, E, F, K and L) or two-way ANOVA (C, H, I, N and O). *P < 0.05; **P < 0.01; ***P < 0.001; ****P < 0.0001. Error bars denote for the s.e.m

We further evaluated the cholesterol metabolic program of tumor infiltrating CD8^+^ T cells. In addition to reduced cholesterol biosynthesis (Fig. S1I–L), we found that LDLR transcription level was decreased in cytotoxic T-lymphocytes (CTLs) upon infiltrating to the tumor microenvironment (Fig. 1A). The reduced surface level of LDLR in tumor infiltrating CD8^+^ T cells was further validated by flow cytometric analysis (Fig. 1B). Meanwhile, we analyzed *LDLR* mRNA level of active CD8^+^ tumor infiltrating lymphocytes (Ki-67^+^ CD8^+^ TILs) and total CD8^+^ TILs according to a scRNA-seq database-tumor immune single-cell Hub (TISCH) which contains an atlas of 76 tumor scRNA-seq datasets (Sun et al., 2020). The result showed that *LDLR* was comparatively lower in total CD8^+^ TILs than that in active CD8^+^ TILs (Fig. S2).

To determine the physiological function of LDLR in CD8^+^ T cells, we isolated the splenic CD8^+^ cells from the *Ldlr*^−/−^ mice in which the T cells showed normal development and homeostasis (Fig. S1N and S1O). When compared with the wild-type CD8^+^ T cells, the *Ldlr*^−/−^ CD8^+^ T cells showed impaired effector function, such as reduced cytokine and granule production, as well as lower clonal expansion, upon stimulation (Fig. 1C and 1D). To further assess the involvement of LDLR in the immune responses of CD8^+^ T cells *in vivo*, we generated antigen-specific LDLR deficient CD8^+^ T cells by crossing OT-I transgenic mice with *Ldlr*^−/−^ mice. We then generated *Ldlr*^−/−^ OT-I CTLs via pulsing splenocytes with OVA_257-264_ peptide (SIINFEKL). We found that LDLR deficiency induced the impairment of immunological synapse formation (Fig. 1E) and cytotoxicity to tumor cells when we cocultured these CTLs with OVA_257–264_ loaded EL4 cells (Figs. 1F and S1M). However, when we transferred the OT-I CTLs to the ovalbumin expressing MC38 tumor (MC38-OVA) burdened mice, we found that *Ldlr* depletion slightly impaired the antitumor activity of CD8^+^ T cells at the late stage. The inconsistent results between *in vitro* and *in vivo* experiments might be due to the rapid decrease of LDLR levels in CD8^+^ T cell while infiltrating to the tumor microenvironment (Fig. 1A and 1B). To overcome the LDLR deficiency induced by tumor microenvironment and further validate the *in vivo* effect of LDLR in CD8^+^ T cells, we over-expressed LDLR in OT-I CTLs using a strong promoter. The adoptive T cell transfer data showed that overexpression of LDLR indeed significantly enhanced the antitumor activity of CD8^+^ T cells *in vivo* (Fig. 1M–O), which is consistent with the *in vitro* data (Fig. 1J–L). Together, these results demonstrate that LDLR intrinsically regulates the immune response and antitumor activity of CD8^+^ T cell.

### The regulation of LDLR on CD8^+^ T cell effector function is not fully dependent on LDL/cholesterol

**Figure 2:**
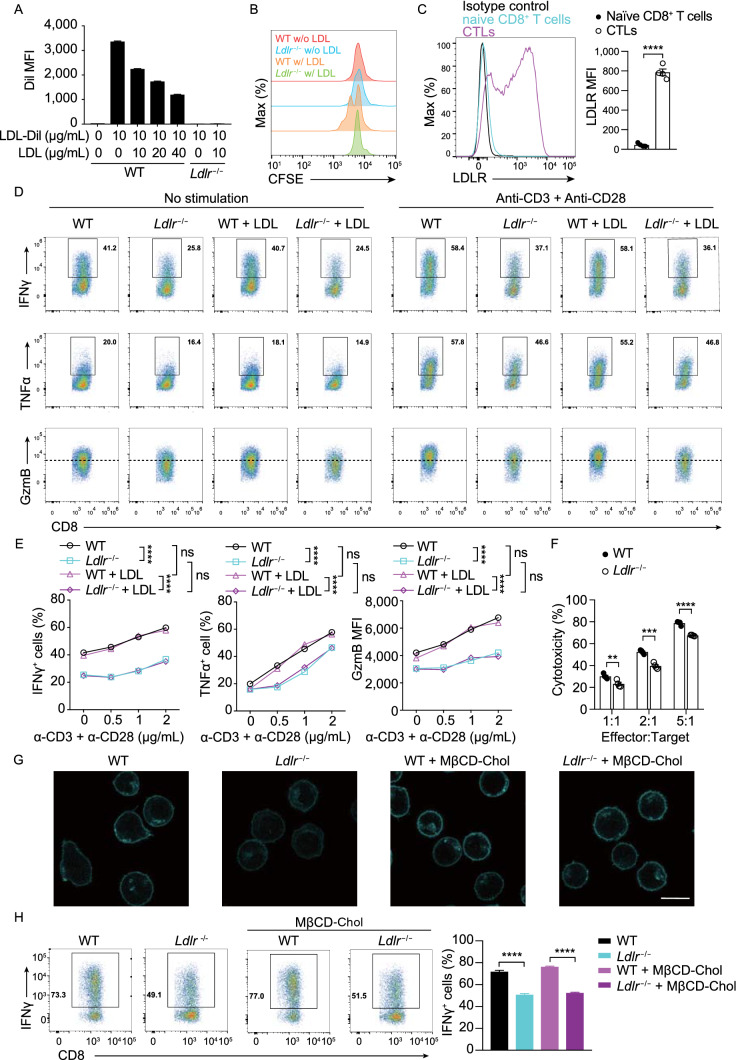
**The regulation of LDLR on CD8**^**+**^**T cell effector function is not fully dependent on LDL/cholesterol**. (A) LDL uptake of activated WT and *Ldlr*^−/−^ CD8^+^ T cells. CD8^+^ T cells were treated with LDL and LDL-Dil at indicated concentrations. The uptake of LDL-Dil was analyzed by flow cytometry. (B) Proliferation of WT and *Ldlr*^−/−^ CD8^+^ T cells was measured by CFSE dilution. Cells were cultured in medium containing lipoprotein-deficient serum (LPDS) with or without the addition of LDL. (C) LDLR expression of naïve CD8^+^ T cells and CTLs was analyzed by flow cytometry, (*n* = 4). (D and E) Cytokine/granule productions of WT and *Ldlr*^−/−^ CTLs. CTLs were generated from the splenocytes of WT and *Ldlr*^−/−^ mice and pretreated in the medium containing LPDS for 4 h, with or without the presence of LDL. The cells were then stimulated with anti-CD3 and anti-CD28 antibodies for 4 h at indicated concentrations in corresponding medium, (*n* = 4). (F) Cytotoxicity of WT and *Ldlr*^−/−^ CTLs. CTLs were pretreated in the medium containing LPDS for 12 h and cocultured with EL4 cells to determine the cytotoxicity, (*n* = 4). (G) Filipin III staining to analyze cellular cholesterol distribution in untreated or MβCD-coated cholesterol treated WT and *Ldlr*^−/−^ CTLs. Scale bar, 10 μm. (H) IFNγ production of WT and *Ldlr*^−/−^ CTLs. Mature CTLs were generated from the splenocytes of WT and *Ldlr*^−/−^ mice and treated with MβCD-coated cholesterol or not. The cells were then stimulated with 1 μg/mL plate-coated anti-CD3 and anti-CD28 antibodies for 4 h, (*n* = 4). Data were analyzed by *t* test (C, F and H) or two-way ANOVA (E). ns, no significance; **P < 0.01; ***P < 0.001; ****P < 0.0001. Error bars denote for the s.e.m

The primary function of the LDLR is to mediate the endocytosis of cholesterol-enriched LDL, which is one of the resources of cellular cholesterol (Jeon and Blacklow, 2005; Go and Mani, 2012). We first measured the LDL uptake in LDLR deficient naïve CD8^+^ T cells upon stimulation. The results exhibited that LDL uptake in CD8^+^ T cells was completely dependent on LDLR (Fig. 2A). Cholesterol is necessary for CD8^+^ T cell priming and clonal expansion (Kidani et al., 2013; Yang et al., 2016; Proto et al., 2018). To evaluate whether LDLR regulates CD8 T cell function is dependent on LDL/cholesterol uptake, we depleted the LDL in the medium and found the proliferation of CD8^+^ T cell was inhibited (Fig. 2B). Moreover, LDL depletion caused the defect of activation and cytokine/granule production of naïve CD8^+^ T cells upon stimulation (Fig. S3). These findings demonstrate that LDLR mediated LDL uptake is essential for naïve CD8^+^ T cell priming and clonal expansion.

After priming and clonal expansion, the resting or naïve CD8^+^ T cells enter into the effector stage in which they are called as activated CTLs. The activated CTLs infiltrate to the tumor microenvironment to kill the tumor cells by recognizing tumor antigens and releasing cytotoxic granule and cytokine (Fu and Jiang, 2018; Hu et al., 2018). To further evaluate the function of LDL/cholesterol which are transported into the cells by LDLR on the CTLs of effector stage, we restimulated the OT-I CTLs with anti-CD3 and anti-CD28 antibodies in lipoprotein deficient serum (LPDS) medium supplemented with or without LDL. In contrast with the data of naïve CD8^+^ T cells, the results in activated CTLs showed that the LDLR deficiency induced impairment of effector function was not relying on LDL supplement (Fig. 2D and 2E), and the *in vitro* CTL killing assay further verified this conclusion (Fig. 2F).

Cholesterol is the dominant component of LDL, and we found that the cholesterol level of LDLR deficient CTLs was decreased, especially in the plasma membrane (Fig. 2G). Previous studies have demonstrated that the plasma membrane cholesterol is involved in T cell activation (Gaus et al., 2005; Wu et al., 2016; Yang et al., 2016). To investigate whether LDLR deficiency-induced impairment of the effector function in CTLs of effector stage is reliant on plasma membrane cholesterol, we artificially increased the plasma membrane cholesterol level of *Ldlr*^−/−^ CTLs by adding Methyl-β-cyclodextrin (MβCD)-coated cholesterol, providing a cholesterol source independent of LDLR expression (Fig. 2G). We then stimulated CTLs with anti-CD3 and anti-CD28 antibodies and evaluated cytokine production by flow cytometry. The results showed that increasing plasma membrane cholesterol did not improve the LDLR deficiency-induced decline of effector function (Fig. 2H). These data indicate that there is a mechanism by which LDLR regulates CTL effector function independent of LDL or cholesterol.

### LDLR interacts with TCR and regulates TCR signaling in CD8^+^ T cells

**Figure 3:**
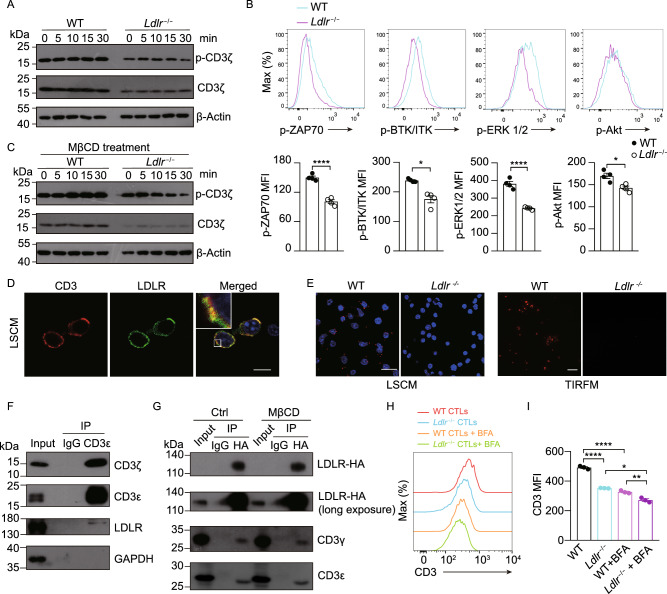
**LDLR interacts with TCR and regulates TCR signaling in CD8**^**+**^**T cells**. (A) Immunoblotting to detect the phosphorylation of CD3ζ of WT and *Ldlr*^−/−^ CTLs. CTLs were stimulated with 1 μg/mL anti-CD3, anti-CD28, anti-Armenian hamster IgG and anti-Syrian hamster IgG for indicated times. (B) Phosphorylation of ZAP70, BTK/ITK, ERK1/2 and Akt of WT and *Ldlr*^−/−^ CTLs. CTLs were stimulated as in (A) for 10 min. Data were analyzed by *t* test (*n* = 4). (C) Immunoblotting to detect the phosphorylation of CD3ζ of MβCD-treated WT and *Ldlr*^−/−^ CTLs. CTLs were stimulated as in (A). (D) Fluorescence staining of CD3 and LDLR in CTLs. DAPI was shown in blue. Scale bar, 10 μm. LCSM, laser confocal scanning microscopy. (E) Proximity Ligation Assay (PLA) analysis of CD3 and LDLR interaction in WT and *Ldlr*^−/−^ CTLs. Confocal images (left panel, scale bar, 20 μm) and TIRFM images (right panel, scale bar, 10 μm) were shown. Red, PLA signals; Blue, DAPI. TIRFM, total internal reflection fluorescence microscopy. (F) CD3ε was immunoprecipitated (IP) in CTLs and its interaction with LDLR was analyzed by immunoblotting. (G) HA-tagged LDLR was overexpressed in EL4 cells. The EL4 cells were treated with MβCD or not and then HA-tagged LDLR was immunoprecipitated with anti-HA antibody. The interaction between LDLR and CD3 was analyzed by immunoblotting. (H and I) WT and *Ldlr*^−/−^ CTLs were treated with BFA (5 μg/mL) or not for 2 h. CD3 expression was analyzed by flow cytometry. Data were analyzed by *t* test (*n* = 3). *P < 0.05; **P < 0.01; ****P < 0.0001. Error bars denote for the s.e.m

We then investigated the underlying mechanisms by which LDLR regulates the effector function of CD8^+^ T cells. Notably, our results showed no significant differences in cytokine and granule production before antibody stimulation (Fig. 1C), the defects induced by *Ldlr* knockout appeared to be observed under stimulation by anti-CD3 antibody. Anti-CD3 antibody stimulation mimics antigen recognition by the TCR and initiates the TCR signaling which is critical for T cell activation and effector function (Riddell and Greenberg, 1990). Thus, we evaluated the effect of LDLR deficiency on TCR signaling. We stimulated *Ldlr*^−/−^ CD8^+^ T cells with anti-CD3 and anti-CD28 antibodies and detected the phosphorylation level of CD3ζ, a subunit of the TCR complex, and downstream signal pathways. The results showed that CD3ζ phosphorylation was inhibited by LDLR deficiency, as compared with the wild-type cells (Fig. 3A). Consequently, the downstream signal pathways were also attenuated by LDLR deficiency (Fig. 3B). Furthermore, the defects of TCR phosphorylation were not altered when we stripped the cholesterol from the plasma membrane via MβCD treatment (Fig. 3C), which suggested that the defects of TCR signaling in *Ldlr*^−/−^ CD8^+^ T cells were not relying on the reduced cholesterol level in the plasma membrane. Previous studies have demonstrated that TCR signaling is influenced by multiple factors, including kinases, phosphatases, the plasma membrane lipids composition and especially the other membrane proteins (Xu et al., 2008; van der Merwe and Dushek, 2011; Stanford et al., 2012; Shi et al., 2013; Alcover et al., 2018). As a cholesterol transporter, LDLR regulated the effector function of CD8^+^ T cells was also independent of plasma membrane cholesterol (Fig. 2H), thus we speculated that LDLR might be directly involved in TCR signaling as a membrane protein.

We next stained CD8^+^ T cells with anti-LDLR and anti-CD3 antibodies to determine the localization of these two proteins on the plasma membrane. Imaging data showed that LDLR colocalizes with CD3 on the plasma membrane of CD8^+^ T cells (Fig. 3D). To further corroborate the interaction between LDLR and TCR complex, we used a proximal ligation assay (PLA) to image the interaction. Confocal imaging data exhibited clear interaction spots in wild-type CD8^+^ T cells, but not in *Ldlr*^−/−^ CD8^+^ T cells (Fig. 3E). Furthermore, total internal reflection fluorescence microscopy (TIRFM) imaging showed the interaction of the LDLR and TCR complex was on the plasma membrane or in the membrane proximal region of CD8^+^ T cells (Fig. 3E). We then used a co-immunoprecipitation (Co-IP) assay to verify the interaction between the CD3 subunits of TCR and LDLR. The results showed that there is indeed an interaction between the CD3 subunits and LDLR, and that this interaction is not influenced by the removal of plasma membrane cholesterol by MβCD treatment (Fig. 3F and 3G).

Additionally, we found that the surface TCR level was reduced in *Ldlr*^−/−^ CD8^+^ T cells (Fig. 3H and 3I). To investigate further, we inhibited plasma membrane protein recycling via treatment with Brefeldin A (BFA) and compared the surface level of TCR between *Ldlr*^−/−^ and wild-type CD8^+^ T cells (Fig. 3H and 3I). These results thus suggest that LDLR may be involved in plasma membrane TCR recycling, thereby regulating TCR signaling and ultimately T cell effector function (Fig. S4).

Together, these data demonstrated that LDLR interacts with the TCR complex and regulates TCR signaling as an immune regulatory membrane protein, not just as an LDL transporter.

### Tumor-derived PCSK9 inhibits the antitumor activity of CD8^+^ T cells

**Figure 4:**
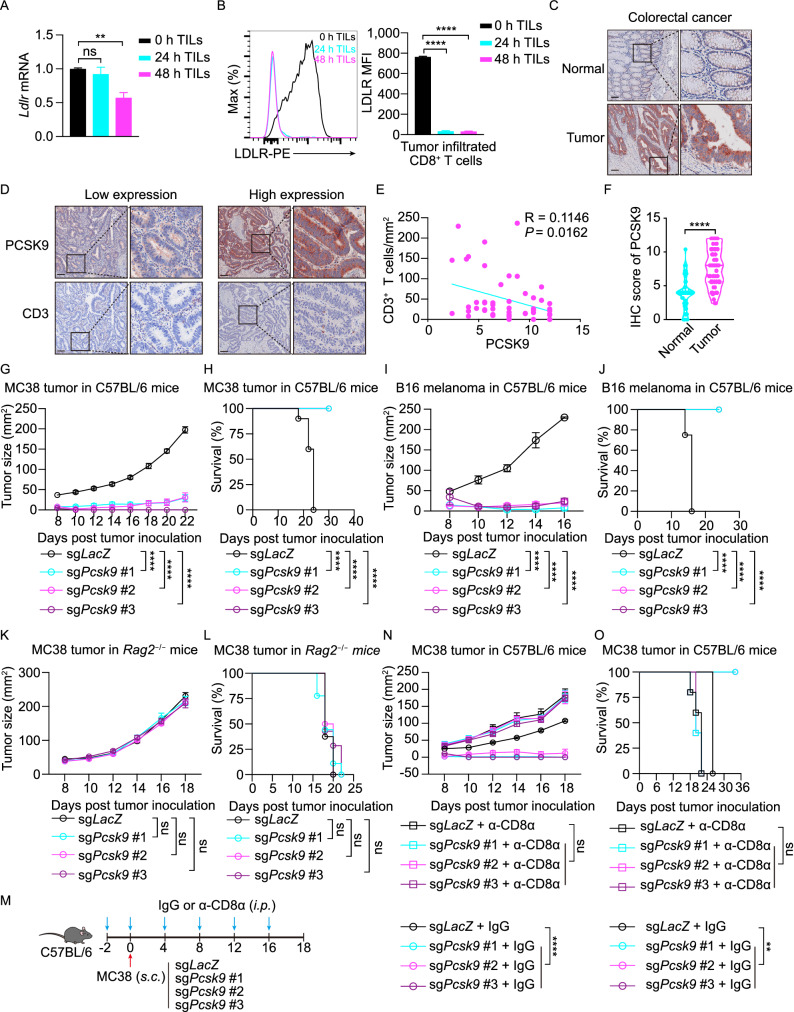
**Tumor-derived PCSK9 inhibits the antitumor activity of CD8**^**+**^**T cells**. (A and B) Transcriptional level (A) and surface protein level (B) of LDLR were assessed in tumor infiltrating CD8^+^ T cells at 0, 24 or 48 h post CTLs adoptive transfer. Data were analyzed by *t* test (*n* = 4–6). (C and F) Human normal colorectal or tumor sections were stained with anti-PCSK9 antibody by immunohistochemistry and the abundance of PCSK9 was assessed in (F). Data were analyzed by *t* test (*n* = 50). (D and E) PCSK9 and CD3 staining were shown in PCSK9 low-expression and high-expression tumors. Pearson correlation coefficient (R) and *P* value (*P*) of PCSK9 expression and CD3^+^ cells infiltration were analyzed in (E). (G and H) Tumor growth (G) and survival (H) of *Pcsk9* knockout MC38 tumor-bearing C57BL/6 mice, (*n* = 10). (I and J) Tumor growth (I) and survival (J) of *Pcsk9* knockout B16F10 melanoma-bearing C57BL/6 mice, (*n* = 7–8). (K and L) Tumor growth (K) and survival (L) of *Pcsk9* knockout MC38 tumor-bearing *Rag2*^−/−^ mice, (*n* = 7–9). (M) Illustration of treatment of MC38 tumor-bearing C57BL/6 mice with IgG or anti-CD8α antibody. (N and O) Tumor growth (N) and survival (O) of *Pcsk9* knockout MC38 tumor-bearing C57BL/6 mice with or without CD8α^+^ cells depletion. Data were analyzed by two-way ANOVA (*n* = 5–6). Scale bar, 120 μm. ns, no significance; **P < 0.01; ****P < 0.0001. Error bars denote for the s.e.m

To further investigate that how the TME inhibits the LDLR level in tumor infiltrating CD8^+^ T cells, we transferred OT-I CTLs to *Rag2*^−/−^ mice with MC38-OVA tumors. Then, we isolated the tumor infiltrating antigen specific CD8^+^ T cells and quantified the mRNA level and cell surface expression of LDLR by qPCR and flow cytometry, respectively. The results showed that cell surface level of LDLR was dramatically decreased at early stage of T cell infiltration, while conversely, the mRNA level remained normal (Fig. 4A and 4B). This finding indicated that there is another pathway that regulates the cell surface level of LDLR besides of transcriptional regulation.

PCSK9, a previously identified LDLR modulator and a therapeutic drug target for treating hypercholesterolemia, has been implicated for regulating LDLR protein level via mediating LDLR internalization and degradation (Abifadel et al., 2003; Maxwell et al., 2005; Cunningham et al., 2007; Zhang et al., 2007; He et al., 2020; Liu et al., 2020). To determine PCSK9 involvement in surface LDLR regulation, we first collected clinical samples of colorectal cancer (CRC), lung cancer and breast cancer to detect PCSK9 expression by immunohistochemistry (IHC). IHC score showed there was higher PCSK9 expression in cancerous regions than that in the adjacent normal region (Fig. 4C, 4F, S5A and S5B). Furthermore, when we detected the CD3 level in the CRC samples, we found there was a significant negative correlation between CD3^+^ T cell infiltration and PCSK9 level (Fig. 4D and 4E). In addition, TCGA data showed that higher PCSK9 level accompanied with worse prognosis (Fig. S6).

To further evaluate the relationship of T cell antitumor activity and PCSK9 expression in tumors, we depleted *Pcsk9* gene expression in a mouse CRC cell line (MC38) and melanoma cell line (B16F10) via CRISPR/Cas9. Then, we transplanted the gene modified tumor cells into wild-type syngeneic mice. The results showed that PCSK9 depletion inhibited tumor progression and greatly extended mice survival time (Fig. 4G–J). Conversely, when we transplanted the MC38 tumor cells to *Rag2*^−/−^ mice which exhibit T cell and B cell deficiency, we found there were no significant differences between the wild-type MC38 and *Pcsk9* knockout MC38 tumor (Fig. 4K and 4L). A similar result was observed by using shRNA to induce *Pcsk9* knockdown in MC38 tumor (Fig. S5D–H), and the tumor infiltrating CD8^+^ T cells in *Pcsk9*-knockdown tumors showed increased antitumor activity (Fig. S5I). Meanwhile, we found the MHC-I (H-2K^b^) and PD-L1 expression in B16F10 melanoma cells were comparable (Fig. S5C). These findings indicated that the lower progression of *Pcsk9* knockout tumor in immunocompetent mice may be attributed to the antitumor activity of adoptive immune cells, like T cells and B cells. As is well known that CD8^+^ T cells are the killer cells for tumor, to validate whether the inhibition of tumor progression by PCSK9 knockout is via CD8^+^ T cells, we used anti-CD8 antibody to deplete CD8^+^ T cells *in vivo*. Our data showed that when CD8^+^ T cells were depleted, there were no significant differences between the wild-type MC38 and the *Pcsk9*-knockout MC38 tumors in the syngeneic immunocompetent mice (Fig. 4M–O). Collectively, these results demonstrate that the tumor derived PCSK9 predominantly inhibits the immune response of CD8^+^ T cells in achieving immune evasion.

Of note, we also investigated the intrinsic effect of PCSK9 on CD8^+^ T cells. We stimulated the splenic naïve CD8^+^ T cells from *Pcsk9*^−/−^ mice with anti-CD3 and anti-CD28 antibodies to detect cytokine and granule production. The results showed PCSK9-deficent CD8^+^ T cells exhibited enhanced effector function (Fig. S7A). Moreover, we found that PCSK9 intrinsically inhibited CD8^+^ T cell function through evaluating the immunological synapse formation and cytotoxicity (Fig. S7B and S7C), as well as the antitumor activity *in vivo* through adoptive T cell transfer assay (Fig. S7D and S7E). These findings indicated that PCSK9 may intrinsically inhibit the antitumor activity of CD8^+^ T cells.

### PCSK9 inhibits CD8^+^ T cell antitumor activity via LDLR and TCR signaling inhibition

**Figure 5:**
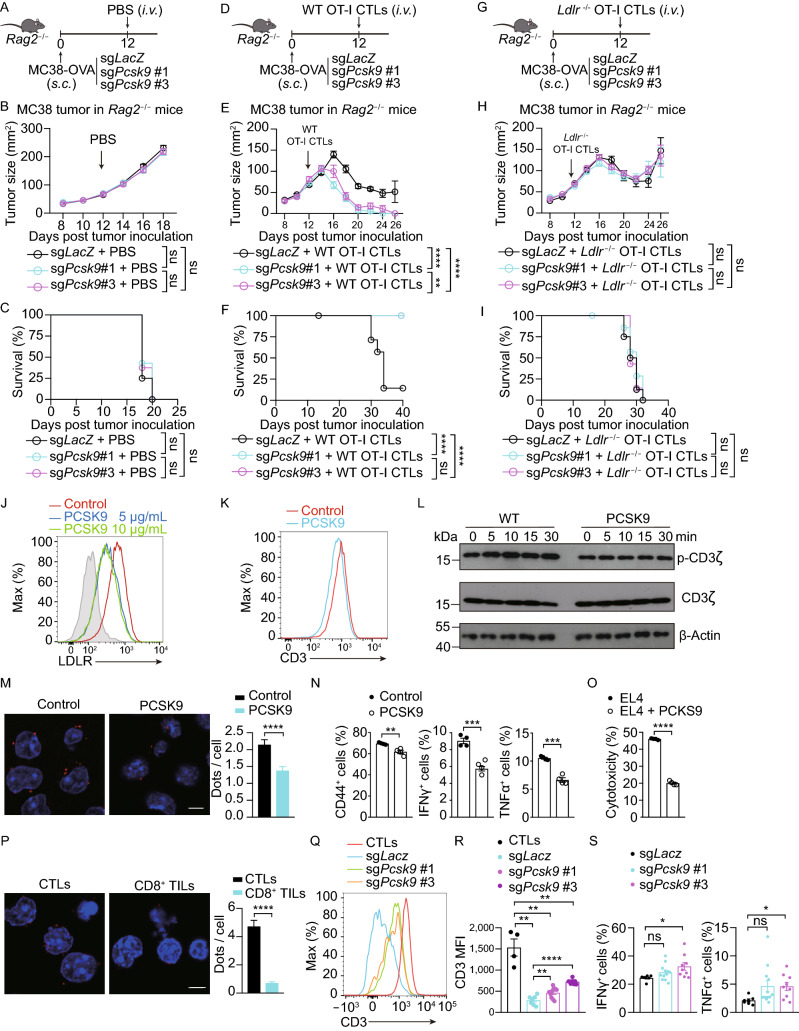
**PCSK9 inhibits CD8**^**+**^**T cell antitumor activity via LDLR and TCR signaling inhibition**. (A) Illustration of adoptive transfer of PBS to MC38-OVA tumor-bearing *Rag2*^−/−^ mice. (B and C) Tumor growth (B) and survival (C) of *Pcsk9* knockout MC38-OVA tumor-bearing *Rag2*^−/−^ mice after adoptive transfer of PBS. (D) Illustration of adoptive transfer of WT CTLs to MC38-OVA tumor-bearing *Rag2*^−/−^ mice. (E and F) Tumor growth (E) and survival (F) of *Pcsk9* knockout MC38-OVA tumor-bearing *Rag2*^−/−^ mice after adoptive transfer of WT CTLs. (G) Illustration of adoptive transfer of *Ldlr*^−/−^ CTLs to MC38-OVA tumor-bearing *Rag2*^−/−^ mice. (H and I) Tumor growth (H) and survival (I) of *Pcsk9* knockout MC38-OVA tumor-bearing *Rag2*^−/−^ mice after adoptive transfer of *Ldlr*^−/−^ CTLs. Data were analyzed by two-way ANOVA in (A–I) (*n* = 7–8). (J) LDLR expression was measured in PCSK9-treated CTLs by flow cytometry. CTLs were treated with PCSK9 protein at indicated concentrations for 6 h. (K) CTLs were treated with 5 μg/mL PCSK9 protein for 6 h. CD3 expression was measured by flow cytometry. (L) Immunoblotting to detect the total and phosphorylation of CD3ζ in control and PCSK9-treated CTLs. CTLs were pretreated with 5 μg/mL PCSK9 protein for 6 h and stimulated with 1 μg/mL anti-CD3, anti-CD28, anti-Armenian hamster IgG and anti-Syrian hamster IgG for indicated times. (M) Proximity Ligation Assay (PLA) analysis of CD3 and LDLR interaction in control or PCSK9-treated CTLs. Scale bar, 5 μm. Red, PLA signals; Blue, DAPI. (N) Activation and cytokine productions of PCSK9 treated activated CD8^+^ T cells. Naïve CD8^+^ T cells were isolated and stimulated with 2 μg/mL anti-CD3 and anti-CD28 antibodies in the presence of PCSK9 protein (5 μg/mL) or not, (*n* = 4). (O) Cytotoxicity of WT CTLs cocultured with PCSK9 overexpressed EL4 cells. PCSK9 was overexpressed in EL4 cells by retrovirus infection. CTLs were cocultured with the EL4 cells to determine the cytotoxicity, (*n* = 4). (P) PLA analysis of CD3 and LDLR interaction in CTLs or CD8^+^ TILs. Scale bar, 5 μm. Red, PLA signals; Blue, DAPI. (Q and R) CD3 surface level was analyzed by flow cytometry in CTLs and TILs isolated from *Pcsk9* knockout MC38-OVA tumors at Day7 post CTLs adoptive transfer, (CTLs, *n* = 4; TILs, *n* = 7–10). (S) IFNγ and TNFα production in isolated TILs of *Pcsk9* knockout MC38-OVA tumors. Data were analyzed by *t* test (M–P, R and S). ns, no significance; *P < 0.05; **P < 0.01; ***P < 0.001; ****P < 0.0001. Error bars denote for the s.e.m

To further investigate the mechanisms how PCSK9 regulates CD8^+^ T cell antitumor activity, we transplanted wild-type MC38-OVA or *Pcsk9*-depleted MC38-OVA cells into *Rag2*^−/−^ mice. We then transferred wild-type OT-I CTLs or *Ldlr*^−/−^ OT-I CTLs into the tumor burdened mice. The results showed that there was no significant difference in tumor progression between the wild-type MC38 tumor and *Pcsk9*-depleted MC38 tumor in *Rag2*^−/−^ mice who did not receive the CTL transfer (Fig. 5A–C). In accord with our earlier findings, the antitumor activity of the wild-type CTLs was significantly higher in the *Pcsk9*-depleted MC38 tumor than that in the wild-type MC38 tumor (Fig. 5D–F). Conversely, when we transferred *Ldlr*^−/−^ OT-I CTLs into the tumor burdened mice, there were no significant differences in tumor progression between the wild-type MC38 tumor and the *Pcsk9*-depleted MC38 tumor (Fig. 5G–I). These findings indicated that the PCSK9-derived inhibition of antitumor activity of CD8^+^ T cell is through LDLR.

Concurrently, we treated CD8^+^ T cells with recombinant mouse PCSK9 protein. These results showed that the surface level of LDLR in CD8^+^ T cells was reduced by PCSK9 treatment (Fig. 5J) and consequently, the plasma membrane TCR level, CD3 phosphorylation and LDLR-CD3 interaction spots were all down-regulated (Fig. 5K–M). Furthermore, we found that PCSK9 treatment inhibited cytokine production of CD8^+^ T cells (Fig. 5N). We then used the *in vitro* killing assay to assess the influence of PCSK9 on CTL cytotoxicity, with PCSK9 over-expressing EL4 cells as the target cells. We found that the overexpression of PCSK9 substantially inhibited the killing efficiency of OT-I CD8^+^ T cells (Fig. 5O). These findings are consistent with the conclusion from LDLR deficient CD8^+^ T cells.

To further evaluate the *in vivo* effects of PCSK9 on TCR, we first detected LDLR-TCR interaction spots with PLA and we found that this interaction is dramatically inhibited in tumor infiltrating CD8^+^ T cells (Fig. 5P). Then, we transplanted wild-type and *Pcsk9*-depleted MC38-OVA cells to *Rag2*^−/−^ mice and transferred OT-I CTLs to the tumor burdened mice. At day 7 post tumor inoculation, we isolated the tumor infiltrating CD8^+^ T cells and performed flow cytometric analysis. The results showed that the TME inhibited the surface level of TCR and the effector function but that PCSK9 depletion alleviated this inhibition (Fig. 5Q–S). Collectively, these data demonstrated that the tumor derived PCSK9 may downregulate LDLR, TCR signaling and effector function of CD8^+^ T cells, thus inhibiting the antitumor activity of CD8^+^ T cells in the TME.

### Inhibiting PCSK9 potentiates the antitumor activity of CD8^+^ T cells

Targeting the PCSK9/LDLR axis has shown clinical potential in treating hypercholesterolemia, multiple drugs, such as evolocumab and alirocumab (Blom et al., 2014), have been approved for clinical use. Herein, we intensively investigated the PCSK9/LDLR axis in the CD8^+^ T cell antitumor immune response. To evaluate whether targeting the PCSK9/LDLR axis possesses clinical cancer treatment potential, we used syngeneic mouse models to evaluate the antitumor effect of PCSK9 inhibitor. The blocking antibodies used, evolocumab and alirocumab, are humanized antibodies. Previous research has found that the binding affinity of evolocumab to mouse PCSK9 (*K*_d_ = 17 nmol/L) is 1000-fold less than its binding affinity to human PCSK9 (*K*_d_ = 16 pmol/L) (Brody and Brody, 2018). Similarly, the binding affinity of alirocumab to mouse PCSK9 (*K*_d_ = 2.61 nmol/L) is 4.5-fold less than the binding affinity to human PCSK9 (*K*_d_ = 0.58 nmol/L) (Kuhnast et al., 2014). Therefore, we used a chemical inhibitor, PF-06446846, which has been demonstrated previously to effectively inhibit mouse PCSK9 expression through slowing down PCSK9 translation, otherwise the blocking antibodies, to testify the antitumor effect by inhibiting PCSK9.

**Figure 6:**
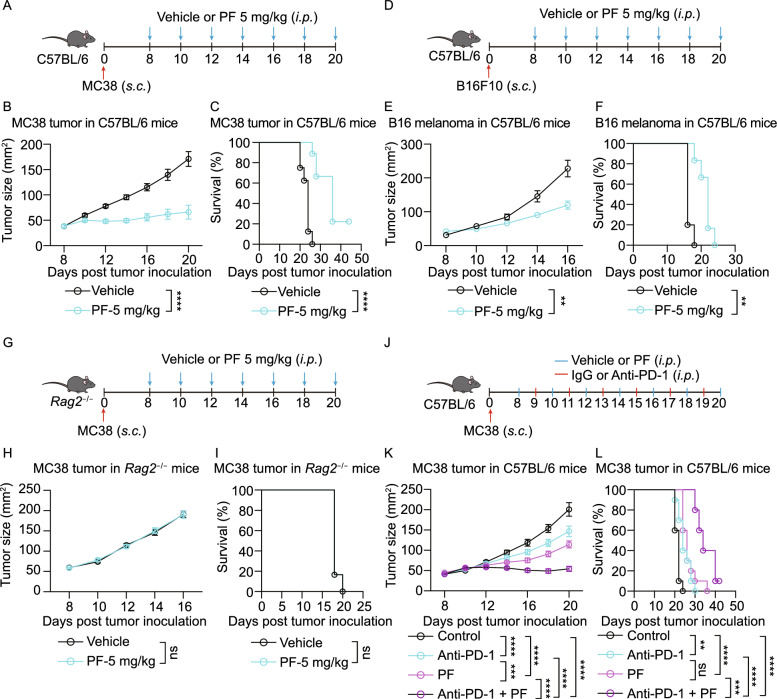
**Inhibiting PCSK9 potentiates the antitumor activity of CD8**^**+**^**T cells**. (A–C) Vehicle or PF-06446846 (5 mg/kg) were injected into MC38 tumor-bearing C57BL/6 mice as illustrated in (A). Tumor growth (B) and survival (C) of MC38 tumor-bearing C57BL/6 mice were shown, (*n* = 8–9). (D–F) Vehicle or PF-06446846 (5 mg/kg) were injected into B16F10 melanoma-bearing C57BL/6 mice as illustrated in (D). Tumor growth (E) and survival (F) of B16F10 melanoma-bearing C57BL/6 mice were shown, (*n* = 8–9). (G–I) Vehicle or PF-06446846 (5 mg/kg) were injected into MC38 tumor-bearing *Rag2*^−/−^ mice as illustrated in (G). Tumor growth (H) and survival (I) of MC38 tumor-bearing *Rag2*^−/−^ mice were shown, (*n* = 6). (J–L) A combined therapy (PF-06446846 and anti-PD-1) or monotherapies (PF-06446846 or anti-PD-1) were utilized for treating MC38 tumors on C57BL/6 mice. Tumor growth (K) and survival (L) of MC38 tumor-bearing C57BL/6 mice were shown, (*n* = 10). Data were analyzed by two-way ANOVA (B, C, E, F, H, I, K and L). ns, no significance; **P < 0.01; ***P < 0.001; ****P < 0.0001. Error bars denote for the s.e.m

First, we assessed the inhibitory effect of PF-06446846 on tumor PCSK9 *in vivo* and found that 7 administrations of a 5 mg/kg dose effectively inhibited PCSK9 expression in MC38 tumors in C57BL/6 mice (Fig. S8A and S8B). We then further evaluated the antitumor effect of PF-06446846 in the syngeneic mouse tumor model, including MC38 and B16 tumors, in which administration of PF-06446846 effectively inhibited tumor progression (Fig. 6A–F). In contrast, there was no analogous antitumor effect with PF-06446846 administration in MC38 tumor burdened *Rag2*^−/−^ mice (Fig. 6G–I). These findings were consistent with those of the *Pcsk9*^−/−^ tumor cells. Furthermore, the *in vitro* CTL killing assay showed that EL4-OVA cells pretreated with PF-06446846 increased the cytotoxicity of OT-I CTLs to the target cells (Fig. S8C). Collectively, these findings indicated that PCSK9 inhibition potentiates the antitumor activity of CD8^+^ T cells.

We then tested a combination therapy of PCSK9 inhibition and immune checkpoint blockade therapy to assess the potential synergistic effect. We treated MC38 tumor burdened C57BL/6 mice, which are immunocompetent syngeneic mice, with PF-06446846 and anti-PD1 antibody (Fig. 6J–L). The results showed that the combination therapy had a stronger tumor suppressive effect than either monotherapy, highlighting that PCSK9 inhibition has potential as a novel cancer immunotherapy strategy.

## Discussion

T cells undergo distinctive metabolic reprograming in different stages, and these metabolic regulations have been demonstrated to play critical roles in immune responses of T cells (Ecker et al., 2018; Geltink et al., 2018; Chapman et al., 2020). As a main component of cellular metabolism, cholesterol metabolism is essential for effective T cell immune responses. But precisely how cholesterol metabolic pathways regulate CD8^+^ T cell function and how metabolic reprogramming regulates CD8^+^ T cell antitumor activity, needs more extensive and comprehensive investigation. Our previous study, and several related studies, have shown that the storage and biosynthesis pathways of cholesterol play important roles in the regulation of the CD8^+^ T cell immune response (Bensinger et al., 2008; Yang et al., 2016). These studies support that CD8^+^ T cells need free cholesterol to support priming and clonal expansion. The TME has been demonstrated as a hypoxia, nutrient restricted environment (Zhang and Ertl, 2016). Can CD8^+^ T cells obtain sufficient cholesterol in the tumor microenvironment to support their effector function and antitumor activity? And if so, how? To answer these questions, we measured the cholesterol/LDL distribution in cancerous and paracancerous normal tissues in mice models and clinical samples from cancer patients. We found that APOB, which is a marker of LDL/cholesterol, showed higher levels in tumor regions compared with normal tissue regions. However, the cellular cholesterol level of tumor infiltrating CD8^+^ T cells was substantially lower than those of peripheral CD8^+^ T cells, suggesting that the cholesterol metabolic pathways might be reprogramed. Further study confirmed this hypothesis, the cholesterol biosynthesis pathway and uptake pathway regulated by LDLR were found to be suppressed in the tumor microenvironment (Fig. S1).

LDLR has been well characterized as a transporter of LDL, and LDLR deficiency has been identified as the cause of high serum LDL, hypercholesterolemia, and other related metabolic dysfunction diseases (Hobbs et al., 1990). The downregulation of LDLR might be a significant factor influencing the cellular cholesterol level of tumor infiltrating CD8^+^ T cells. Our *in vitro* and *in vivo* experiments demonstrated that LDLR is in fact necessary for CD8^+^ T cell antitumor immunity (Fig. 1). When we assessed the function of LDL/cholesterol in CD8^+^ T cells, we found LDL/cholesterol is essential for CD8^+^ T cell priming and proliferation, but not for effector function, particularly in activated CTLs (Fig. 2). This phenomenon might be due to well-established reprogramming of cholesterol metabolism in CTLs in which the enhanced biosynthesis may provide majority of the cholesterol for proliferation and other related functions of CD8^+^ T cells (Fig. S1I). In the further study to explore the mechanism that why LDLR regulates the effector function is independent of LDL in CTLs, we found that LDLR interacts with the TCR on the plasma membrane of CD8^+^ T cells. This interaction favors TCR signaling and the effector function of CD8^+^ T cells. LDLR deficiency appears to inhibit TCR recycling to the plasma membrane as well as TCR signaling (Fig. 3). Taken together, we found a noncanonical function of LDLR, which functions as a membrane protein to regulate the other receptors on the plasma membrane, not just as an LDL/cholesterol transporter. This finding indicates that LDLR could regulate other membrane proteins and may be involved in more physiological functions in a variety of cell types.

After elucidating the critical role of LDLR, the next question was how does the TME inhibit LDLR expression in CD8^+^ T cells? Generally, protein expression can be inhibited at two levels: the transcriptional level and the post-transcriptional level. T cell activation by antigen stimulation can upregulate *Ldlr* mRNA level (Yang et al., 2016), which is consistent with the finding that there are multiple transcriptional-factor binding sites for AP1 (c-Jun/c-Fos), NFκB and NFAT which are regulated by TCR signaling in T cells when we predicted the cis-element of *Ldlr* promoter by bioinformatic analysis. Moreover, T cell activation may downregulate IDOL, which is the E3 ligase of LDLR and mediates LDLR ubiquitination and degradation (Zelcer et al., 2009; Yang et al., 2016). In the past years, PCSK9, which has been shown to be a negative modulator of LDLR, has been utilized as a clinical drug target for treating hypercholesterolemia (Stein et al., 2013; Raal et al., 2015; Raal et al., 2017). We found that PCSK9 was highly expressed in the tumor region of patients and that T cell infiltration was negatively correlated with the PCSK9 level (Fig. 4A–F). One research published recently also showed that PCSK9 can act as an efficient target in cancer immunotherapy (Liu et al., 2020). They found PCSK9 could interact with MHC-I and disrupt its recycling to the cell surface in tumor cells. In our experiments, there were no significant differences of the expression of MHC-I and PD-L1 in *Pcsk9* knockout B16F10 cells *in vitro*. Furthermore, our findings suggest that tumor cell derived PCSK9 may downregulate the surface LDLR level in CD8^+^ T cells (Fig. 5J–O), thereby inhibiting the antitumor activity of CD8^+^ T cells. Given that the LDLR level of CD8^+^ T cells was downregulated during early stage of infiltration, while the transcription of *Ldlr* was not altered (Fig. 4A and 4B). And in combination with the finding that LDLR may directly regulate TCR signaling (Fig. 3), we speculate that the TME derived PCSK9 may be the trigger of LDLR downregulation and consequently induce the immune suppression of CD8^+^ T cells. This speculation was confirmed in the syngeneic mouse tumor model, in which the depletion of tumor PCSK9 alleviated the immune suppression on CD8^+^ T cells (Figs. 4G–O, 5R and 5S).

Moreover, when we examined the intrinsic function of PCSK9 in CD8^+^ T cells, we found that PCSK9 intrinsically inhibited the effector function of CD8^+^ T cells, with the PCSK9 knockout CD8^+^ T cells exhibiting higher antitumor activities (Fig. S7), which indicates that the simultaneous inhibition of PCSK9 expression in tumor cells and CD8^+^ T cells may be a therapeutic approach to potentiate CD8^+^ T cell antitumor immunity.

Targeting metabolic reprogramming has been demonstrated as a potential method for cancer immunotherapy (Sukumar et al., 2013; Dugnani et al., 2017; Kishton et al., 2017). To further assess the clinical potential of inhibiting PCSK9, we used a chemical inhibitor of PCSK9, PF-06446846, which has been proved to inhibit PCSK9 translation (Lintner et al., 2017). This inhibitor successfully improved antitumor activity in a syngeneic mouse tumor model and when it was used in combination with anti-PD-1 antibody, the antitumor effect was further enhanced (Fig. 6). These findings further support that targeting the metabolic pathway of cholesterol is a potential approach for cancer immunotherapy.

**Figure 7:**
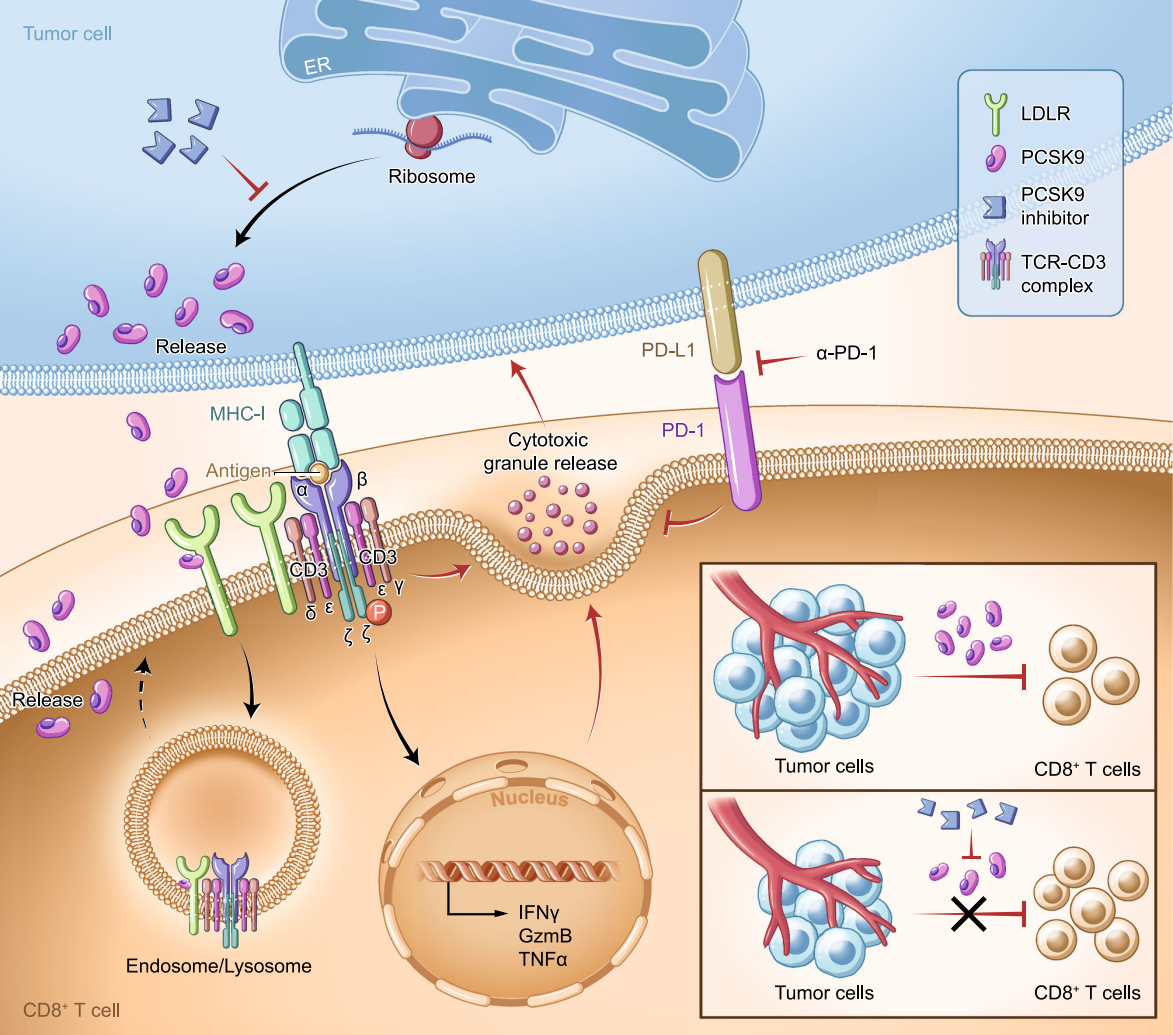
**Schematic illustration of the regulatory roles of PCSK9/LDLR/TCR axis on the antitumor activity of CD8**^**+**^**T cells**. LDLR is essential for the CD8^+^ T cell priming, clonal expansion, and effector function. Besides the canonical role on LDL uptake, LDLR can interact with the CD3 subunits of TCR complex and regulate TCR recycling and signaling. As a negative regulator of LDLR, PCSK9 can bind to LDLR and prevent the recycling of LDLR and TCR to the plasma membrane. Inhibition of PCSK9 can enhance the antitumor activity of CD8^+^ T cells, indicating PCSK9/LDLR/TCR axis as a potential target for cancer immunotherapy

In summary, we have demonstrated that LDLR functions as a critical immune regulatory receptor for CD8^+^ T cells in the tumor microenvironment. Furthermore, we reported a novel mechanism for LDLR activity, whereby it interacts with TCR to regulate TCR signaling, ultimately impacting CD8^+^ T cells effector function. Further investigation revealed that tumor cell derived PCSK9 is the critical factor for immune suppression of CD8^+^ T cells in the tumor microenvironment (Fig. 7). Collectively, our findings highlight that the PCSK9-LDLR-TCR axis is the “metabolic immune checkpoint” of the tumor microenvironment and that targeting this pathway holds great potential in cancer immunotherapy.

## Methods and materials

### Patients and clinical specimens

The paraffin embedded tissues of colorectal carcinoma (CRC) tissues, adjacent non-carcinoma tissues (ANT), lung cancer tissues and breast cancer tissues were obtained from the tissue bank of the Department of Pathology, Nanfang Hospital, Southern Medical University. Samples were collected from colorectal cancer, lung cancer and breast cancer that had been clinically diagnosed as cancer. The study protocols concerning human subjects are consistent with the principles of the declaration of Helsinki. The study was approved by the Clinical Research Ethics Committee of Southern Medical University.

### Mice

C57BL/6 mice, *Rag2*^–/–^ mice, *Ldlr*^–/–^ mice, *Pcsk9*^–/–^ mice and OT-I TCR transgenic mice were originally purchased from the Jackson Laboratory. Through mouse crossing, *Ldlr*^–/–^ OT-I mice and *Pcsk9*^–/–^ OT-I mice were obtained, and the genotypes were validated by PCR. All mice used in this study are maintained in specific pathogen-free conditions. All animal experiments used mice were randomly allocated to specific groups with matched age and sex. All animal experiments were approved by the Ethics Committee on Use and Care of Animals of Southern Medical University.

### Reagents and antibodies

For flow cytometric analysis, anti-CD3ε (145-2C11), anti-CD8 (53-6.7), anti-CD44 (IM7), anti-CD45 (30-F11), anti-IFNγ (XMG1.2), anti-granzyme B (NGZB), anti-TNF-α (MP6-XT22), anti-p-ZAP70/Syk (Tyr319, Tyr352) (n3kobu5), anti-p-BTK/ITK (Tyr551, Tyr511) (M4G3LN), anti-p-Akt (Ser473) (SDRNR) and anti-p-Erk (Thr202, Tyr204) (MILAN8R) were purchased from Thermofisher. Anti-mouse LDLR (101) was purchased from Sino Biological Inc. For Western blot analysis, anti-β-actin, anti-GAPDH, anti-CD3ε, anti-CD3γ and anti-CD3ζ were from Santa Cruz Biotechnology. Anti-p-CD3ζ (Tyr142) was from Abcam. Anti-HA was from Sigma. For immunohistochemistry analysis, anti-Apolipoprotein B (Abcam), anti-PCSK9 (Sino Biological) and anti-CD3 (SP7, Abcam) were purchased from indicated companies. For immunofluorescence and PLA staining, anti-LDLR was from Lifespan. Anti-CD3 was from Genetex. Anti-CD3ε was from Bio X Cell. Filipin III was from Cayman. PF-06446846 was from MedChemExpress. For tissue infiltrated T cells isolation, Type IV Collagenase was from Gibco. DNase I was from Applichem. Hyaluronidase was from Sigma. Percoll was from GE. Anti-CD3ε (145-2C11, Bio X Cell) and anti-mouse CD28 (37.51, Bio X Cell) were used for T cell activation. OVA_257–264_ peptide (SIINFEKL) was from ChinaPeptides Co. PCSK9 protein was purchased from ACROBiosystems. Celltrace CFSE, Celltracker Deep Red and Cell proliferation Dye eFluor 450 were from Invitrogen. Methyl-beta-cyclodextrin (MβCD) and MβCD-coated cholesterol were purchased from Sigma.

### Cell lines

MC38 cells were provided by JENNIO Biological Technology (Guangzhou, China). B16F10 and EL4 cells were originally obtained from the American Type Culture Collection (ATCC). All cells were proved mycoplasma-free. MC38, B16F10 and 293T cells were maintained in DMEM (Gibco) and EL4 cells were in RPMI-1640 (Gibco) medium respectively, supplemented with 10% FBS and 1% penicillin-streptomycin. Cells were cultured at 37 °C in a humidified atmosphere containing 95% air and 5% CO_2_. MC38-OVA and B16F10-OVA cells were generated by lentivirus infection and OVA^+^ cells were sorted by flow cytometry.

### PCSK9 knockdown and knockout cell lines

To generate PCSK9 knockdown cell lines, lentiviruses were produced by transfecting 293T cells with pLKO.1-GFP, psPAX2 and VSV-G plasmids. MC38 cells were infected with pLKO.1 shRNA lentivirus and GFP^+^ cells were selected by Fluorescence-activated Cell Sorting. Knockdown efficiency was determined by QPCR. ShRNA sequences against *Pcsk9* were as follows: sh*Pcsk9* #1: 5′-GCTGATCCACTTCTCTACC-3′; sh*Pcsk9* #2: 5′-CAGAGGCTACAGATTGAAC -3′.

To generate PCSK9 knockout cells, lentiviruses were produced by transfecting 293T cells with Lenti-CRISPR-V2, psPAX2 and VSV-G plasmids. MC38 and B16F10 cells were infected with lentivirus and GFP^+^ cells were selected by Fluorescence-activated Cell Sorting. SgRNA sequences targeting mouse *Pcsk9* were as follows: sg*Pcsk9* #1, 5′-GCTGATGAGGCCGCACATG-3′; sg*Pcsk9* #2, 5′-CTACTGTGCCCCACCGGCGC-3′; sg*Pcsk9* #3, 5′- ACTTCAACAGCGTGCCGG-3′, SgRNA sequence targeting LacZ: 5′-GCGAATACGCCCACGCGAT-3′.

### Flow cytometric analysis

Anti-mouse CD16/32 antibody was used to block non-specific binding with Fc receptors before all surface staining. For surface staining, cells were collected and staining with antibodies at 4 °C for 30 min. For cytokine staining, cells were stimulated with Brefeldin A (5 µg/mL, invitrogen) for 4 h before cells were harvested for analysis. Before intracellular staining and phosphorylation staining, harvested cells were stained the surface protein and then fixed with 4% PFA for 5 min at RT. Then the cells were permeabilized with 0.1% Triton X-100 for 5 min at RT. Then the cells were stained with specific antibodies for 1 h at 4 °C. Flow cytometric data were analyzed with a SONY SA3800 flow cytometer and FlowJo software (Treestar).

### Immunohistochemistry

Human tissue samples and mouse tumor tissues were embedded with paraffin and sectioned longitudinally at 5 µm. All tissue sections were de-waxed and rehydrated and then antigens were retrieved with 10 mmol/L sodium citrate (pH 6.0) in a pressure cooker. Incubated sections in 0.3% H_2_O_2_ for 30 min for blocking endogenous peroxidase activity. The slides were blocked with goat serum and then incubated with anti-human or mouse PCSK9, anti-human ApoB and anti-human CD3 antibodies at 4 °C overnight. Then the slides were incubated with a goat anti- IgG HRP antibody and developed with 3-amino-9-ethylcarbazole (AEC) and counterstained with hematoxylin. Images were captured by digital slides scanner (KF-PRO-120). Immunohistochemical results were scored in accordance with immunoreactive score (IRS) standards proposed by Remmele and Stegner. IRS = SI (staining intensity) × PP (percentage of positive cells). Negative PP, 0; 10% PP, 1; 10%–50% PP, 2; 51%–80% PP, 3; and >80% PP, 4. Negative SI, 0; Mild SI, 1; Moderate SI, 2; Strongly positive SI, 3. Images were scored independently by two pathologists who were blinded to patient information.

### Real time RT-PCR

Total RNA was extracted with TRIzol reagent (Thermofisher). cDNA was synthesized with the Hiscript III RT Supermix for qPCR Kit (Vazyme) according to the manufacturer’s instructions. Real-time quantitative PCR using gene specific primers (5′-3′): *18s* (forward, TTGATTAAGTCCCTGCCCTTTGT; reverse, CGATCCGAGGGCCTCACTA); *Ldlr* (forward, TGACTCAGACGAACAAGGCTG, reverse, ATCTAGGCAATCTCGGTCTCC); *Srebf1* (forward, GCAGCCACCATCTAGCCTG; reverse, CAGCAGTGAGTCTGCCTTGAT); *Srebf2* (forward, GCAGCAACGGGACCATTCT; reverse, CCCCATGACTAAGTCCTTCAACT); *Acaca* (forward, ATGGGCGGAATGGTCTCTTTC; reverse, TGGGGACCTTGTCTTCATCAT); *Fasn* (forward, GGAGGTGGTGATAGCCGGTAT; reverse, TGGGTAATCCATAGAGCCCAG); *Hmgcs* (forward, AACTGGTGCAGAAATCTCTAGC; reverse, GGTTGAATAGCTCAGAACTAGCC); *Hmgcr* (forward, AGCTTGCCCGAATTGTATGTG; reverse, TCTGTTGTGAACCATGTGACTTC); *Sqle* (forward, ATAAGAAATGCGGGGATGTCAC; reverse, ATATCCGAGAAGGCAGCGAAC); *Idol* (forward, TGCAGGCGTCTAGGGATCAT; reverse, GTTTAAGGCGGTAAGGTGCCA); *Abca1* (forward, AAAACCGCAGACATCCTTCAG; reverse, CATACCGAAACTCGTTCACCC); *Abcg1* (forward, CTTTCCTACTCTGTACCCGAGG; reverse, CGGGGCATTCCATTGATAAGG); *Soat1* (forward, GAAACCGGCTGTCAAAATCTGG; reverse, TGTGACCATTTCTGTATGTGTCC); *Soat2* (forward, ACAAGACAGACCTCTTCCCTC; reverse, ATGGTTCGGAAATGTTCACC); *Nceh* (forward, TTGAATACAGGCTAGTCCCACA; reverse, CAACGTAGGTAAACTGTTGTCCC); *Ifng* (forward, ATGAACGCTACACACTGCATC; reverse, CCATCCTTTTGCCAGTTCCTC); *Pcsk9* (forward, GAGACCCAGAGGCTACAGATT; reverse, AATGTACTCCACATGGGGCAA). All PCR reactions were conducted on a QuantStudio real-time PCR system (Thermo Fisher) in triplicates. Gene expression was normalized to *18s*.

### CD8^+^ T cell isolation and activation

Naïve CD8^+^ T cells were isolated from mouse spleen by a EasySep Mouse Naïve CD8^+^ T cell Isolation Kit (Stem Cell). Then the cells were stimulated with plate-coated anti-CD3 and anti-CD28 antibodies at indicated concentration for indicated times.

### CTLs generation

OT-I mouse splenocytes were harvested and homogenized using sterile techniques. Red blood cells were then lysed with ACK buffer for 5 min at RT. The splenocytes were pelleted and resuspended at 1 × 10^6^ per milliliter in RPMI-1640 medium with 10% FBS, 1% penicillin-streptomycin, 2-mercaptoethanol and supplemented with 10 nmol/L OVA_257–264_ peptide and 10 ng/mL human recombinant interleukin-2 (Peprotech) for 3 days. Then the cells were cultured in fresh medium containing 10 ng/mL IL-2 for 2 more days to do the subsequent experiments.

### Measurement of CD8^+^ T cell proliferation

Isolated naïve T cells were labeled with 0.4 µmol/L CFSE in PBS for 10 min at RT. Then the cells were washed with PBS for 3 times. The cells were stimulated with anti-CD3 and anti-CD28 antibodies (1 µg/mL) for 48 h or 72 h. The cells were collected and stained with anti-CD8 antibody. Then the CFSE fluorescence was detected by flow cytometry.

### Measurement of the cytotoxicity of CTLs

To measure the cytotoxicity of CTLs, EL4 cells were pulsed with 10 nmol/L OVA_257–264_ for 30 min at 37 °C. Then the antigen-pulsed EL4 cells were washed with PBS and then labeled with 1 µmol/L CellTracker Deep Red (CTDR) in serum-free medium for 15 min at 37 °C in dark. Meanwhile, EL4 cells labeled with 0.5 µmol/L CFSE in PBS for 10 min at RT in dark. After washing EL4 cells with PBS for 3 times, CTDR labeled and CFSE labeled EL4 cells were mixed at the ratio of 1:1 in the killing medium (RPMI 1640, 2% FBS). CTLs were added into the plate at the indicated ratio, respectively. After 4 h, the cytotoxic efficiency was measured by quantifying the value of one minus the ratio of CTDR/CFSE ratio in cytotoxic group to non-cytotoxic group.

### Measurement of the immune synapse formation of CTLs

To measure the immune synapse formation between CTL and EL4 cells, EL4 cells were pulsed with 10 nmol/L OVA_257–264_ and labeled with CTDR. CTLs were labeled with CFSE. EL4 cells and CTLs were mixed at the ratio of 1:1 and co-cultured for 30 min at 37 °C. The cells were fixed and harvested for flow cytometric analysis and the percentage of CTDR and CFSE double positive cells were quantified.

### LDLR overexpression in CTLs

LDLR CDS sequence was constructed into pMxs plasmid. Retrovirus was generated by transfecting platE cells with pMxs or pMxs-LDLR plasmid. The supernatant containing the retrovirus was collected. To overexpress LDLR in CTLs, OT-1 CTLs were generated and cultured for 1 day. Then the cells were spin-infected with the retrovirus for 2 h at 2,000 rpm with 10 ng/mL IL-2 and 10 µg/mL polybrene. Spin-infection was repeated at day 2. LDLR overexpressed cells were isolated by Fluorescence-activated Cell Sorting and cultured in RPMI 1640 complete medium in the presence of 10 ng/mL IL-2.

### Mouse models for colorectal cancer and melanoma

MC38, MC38-OVA or B16F10 cells were washed with PBS and filtered through a 40 µm strainer. Before tumor cells were inoculated, age and sex matched mice (6–8 weeks) were narcotized and shaved first, then 1 × 10^6^ MC38, MC38-OVA cells or 4 × 10^5^ B16F10 cells were subcutaneously injected into the dorsal part of mice. From day 6–10, tumors size was measured every 2 days, and animal survival rate was recorded every day. Tumor size was calculated as length × width. Mice will be euthanized when the tumor size was larger than 225 mm^2^ (15 mm × 15 mm) for ethical consideration.

### Adoptive T cell transfer

MC38-OVA cells (1 × 10^6^) were injected subcutaneously into *Rag2*^–/–^ mice at age 6–8 weeks. On day 12, tumor-bearing mice with similar tumor size were randomly divided into specific groups and respectively received PBS, wild-type OT-I CTLs, *Ldlr*^–/–^ OT-I CTLs, *Ldlr* OE OT-I CTLs or *Pcsk9*^–/–^ OT-I CTLs (1 × 10^6^ or 5 × 10^5^) intravenously injection. Tumor size was calculated as length × width every 2 days and animal survival was measured every day from day 8. When the tumor size was larger than 225 mm^2^, the mice were euthanized for ethical consideration.

### Depletion of CD8^+^ T cells *in vivo*

MC38 cells (1 × 10^6^) were inoculated subcutaneously into C57BL/6 mice at 6–8 weeks. Two days before tumor inoculation, 200 µg/mL of α-CD8 depletion antibody (2.43, Bio X Cell) or rat IgG (2A3, Bio X Cell) were intraperitoneally injected into indicated group. Subsequently, α-CD8 depletion antibody or rat IgG were injected for every 4 days.

### Treatment of cancer with PF-06446846, anti-PD-1 antibody or PF-06446846 plus anti-PD-1 antibody *in vivo*

Tumor-bearing mice with similar tumor size were randomly divided into different groups and received PBS, anti-PD-1 antibody (RMP1-14, Bio X Cell, 100 µg per injection), PF-06446846 (5 mg/kg) or anti-PD-1 antibody plus PF-06446846 injection intraperitoneally every 2 days, respectively. PF-06446846 was injected 7 times from day 8 and anti-PD-1 was injected 6 times from day 9. The tumor size and survival were measured as mentioned above.

### Tumor infiltrating lymphocytes (TILs) isolation and analysis

CTLs adoptively transferred *Rag2*^−/−^ mice or tumor-bearing C57BL/6 mice were anesthetized and sacrificed, tumor tissues were dissected and cut into pieces and digested in RPMI 1640 medium containing collagenase VI (210 U/mL), DNase I (100 U/mL) and hyaluronidase (0.5 mg/mL) for 30 min at 37 °C. The dissociated cells were passed through a 70 µm strainer. The filtered cells were centrifuged at 50 ×*g* for 1 min. Then the supernatant was removed to a new tube to centrifuge at 1000 ×*g* for 10 min. Resuspended cells for density gradient centrifugation with 40% Percoll and 70% Percoll. Harvest the interphase of gradient and spin at 1000 ×*g* for 5 min. The isolated tumor infiltrated lymphocytes were then used in the subsequent experiments. To measure the cytokine production of isolated TILs, the cells were stimulated with 50 ng/mL PMA, 1 µmol/L ionomycin and 5 µg/mL BFA for 4 h at 37 °C.

### CD8^+^ T cells isolation

Isolate CD8^+^ T cells in tumor infiltrated lymphocytes was based on EasySep^TM^ Release Mouse Biotin Positive Selection Kit (Stemcell). In brief, tumor infiltrated lymphocytes were resuspended in 500 µL (5 × 10^7^), added Fc blocker and biotin labeled anti-mouse CD8 (53–6.7) antibody and incubated for 15 min at RT. Washed cells with isolation buffer and centrifuge for 5 min at 400 ×*g*. Added selection cocktail and incubated for 15 min at RT. Then RapidSpheres beads were added into incubation system for 10 min at RT under rolling and tilting. After incubating, add isolation buffer and magnetically select beads-bound CD8^+^ T cells. Washed beads-bound CD8^+^ T cells for 3 times and obtain pure beads-bound CD8^+^ T cells.

### Filipin III staining

Isolated tumor infiltrating T cells were washed with PBS for 3 times. Then load cells on the glass dish and incubate at RT for 10 min. Add 4% paraformaldehyde (PFA) and 0.05% glutaraldehyde to fix cells at RT for 10 min. Wash cells with PBS for 3 times and then stain Filipin III at the concentration of 50 µg/mL for 2 h at RT. Cells were washed for 8 times and images were collected using Zeiss (LSM880, AxioObserver) confocal microscope and analyzed using Image J software.

### Modulation of the plasma membrane cholesterol level by MβCD and MβCD-coated cholesterol

To deplete cholesterol from the plasma membrane, CD8^+^ T cells were washed with PBS for two times and then incubated with 1 mmol/L MβCD at 37 °C for 15 min. The cells were then washed three times with PBS.

To add cholesterol to the plasma membrane, CD8^+^ T cells were washed with PBS for two times and then incubated with 10 μg/mL MβCD-coated cholesterol at 37 °C for 15 min. The cells were then washed three times with PBS.

### PCSK9 and PF-06446846 treatment

Isolated naïve CD8^+^ T cells from the spleen were stimulated with anti-CD3 and anti-CD28 antibodies in the presence of PCSK9 (5 μg/mL or 10 μg/mL) for 24 h and cytokine production were then determined.

EL4 and EL4-OVA cells were pretreated with PF-06446846 (5 μmol/L or 10 μmol/L) for 24 h and then cocultured with CTLs for 12 h. The cytotoxic efficiency was measured by flow cytometry.

### Immunofluoresence detection of co-localization

CTLs were harvested and placed in glass bottom cell culture dish and fixed with 4% PFA. After blocking the non-specific binding sites with goat serum for 30 min at RT, the cells were incubated with anti-LDLR (Lifespan) and anti-CD3 (Genetex) primary antibodies for 12 h at 4 °C. Then the cells were stained with Alexa 488-conjugated goat anti-rabbit IgG and Alexa Fluor Plus 555-conjugated donkey anti-mouse IgG for 2 h at 4 °C after washing with PBS. Before imaging, the cells were sealed with In Situ Mounting Medium with DAPI (Sigma). Images were collected using Zessi (LSM880, AxioObserver) confocal microscope.

### Measurement the interaction of LDLR and CD3 by proximity ligation assay (PLA)

PLA allows for endogenous detection of protein interaction. We detect the interaction of LDLR and CD3 according to Duolink PLA Fluorescence protocol (Sigma). Cells were fixed with 4% PFA. Block non-specific signal by adding Duolink Blocking Solution and incubate for 60 min at 37 °C. After blocking, add the anti-LDLR and anti-CD3 primary antibodies and incubated for 12 h at 4 °C. Then two PLA probes were diluted and added to the samples and incubated for 60 min at 37 °C. Prepare ligation and amplification buffer to ligate the fluorescence probe and amplify the signal. Mount the samples with *in situ* Mounting Medium with DAPI (Sigma). The images were captured with Olympus FV1000 or Zess LSM880 confocal microscope, and analyzed with Image J software.

The TIRF-imaging was performed on Nikon N-SIM + N-STORM microscope with a TIRF 100× oil immersion lens. Adjusted the oblique incidence excitation to the appropriate TIRF angle to capture images.

### Co-immunoprecipitation and Western blot analysis

EL4 cells were lysed in Nonidet P-40 lysis buffer and CTLs were lysed in 0.25% Digitonin lysis buffer (50 mmol/L Tris-HCl, pH 7.4, 155 mmol/L NaCl, 5 mmol/L EDTA, 2 mmol/L Na_3_VO_4_, 20 mmol/L NaF, supplemented with complete protease inhibitor cocktail and phosphatase inhibitor cocktail). The target protein was immunoprecipitated with corresponding antibody and by Pierce™ Co-Immunoprecipitation Kit (Thermo Fisher) according to the manufacturer’s instructions.

For Western blot analysis, proteins were separated by SDS-PAGE and transferred to polyvinyl difluoride (PVDF) membrane. Proteins were then probed with specific primary antibodies followed by secondary antibodies conjugated with horseradish peroxidase (HRP).

### Statistical analysis

TCGA gene expression and survival data were acquired via UCSC Xena browser (https://xenabrowser.net/) on Aug 9, 2020. The PCSK9 expression data of primary solid tumor samples and the corresponding clinical phenotype and survival data were analyzed by R software (R-3.6.3-win). LDLR expression in TILs of cancer patients was acquired from a scRNA-seq database-Tumor Immune Single-cell Hub (TISCH) (Sun et al., 2020).

Statistical parameters are all shown in figure legends. Statistical analysis was performed using nonparametric two-tailed *t* test or two-way ANOVA in GraphPad Prism. The survival data were analyzed by using Log-rank (Mantel-Cox) test. Unless specially described, error bars stand for standard error of the mean. *P < 0.05; **P < 0.01; ***P < 0.001; ****P < 0.0001.

## Supplementary Information

Below is the link to the electronic supplementary material.

Supplementary material 1 (PDF 1106 kb)
