# Correction to: Potentiating CD8^+^ T cell antitumor activity by inhibiting PCSK9 to promote LDLR-mediated TCR recycling and signaling

**DOI:** 10.1007/s13238-021-00833-y

**Published:** 2021-09-07

**Authors:** Juanjuan Yuan, Ting Cai, Xiaojun Zheng, Yangzi Ren, Jingwen Qi, Xiaofei Lu, Huihui Chen, Huizhen Lin, Zijie Chen, Mengnan Liu, Shangwen He, Qijun Chen, Siyang Feng, Yingjun Wu, Zhenhai Zhang, Yanqing Ding, Wei Yang

**Affiliations:** 1 Shunde Hospital, Southern Medical University (The First People’s Hospital of Shunde), Foshan, 528308 China; 2 Guangdong Provincial Key Laboratory of Molecular Oncologic Pathology, Department of Pathology, School of Basic Medical Sciences, Southern Medical University, Guangzhou, 510515 China; 3 Department of Pathology, Nanfang Hospital, Southern Medical University, Guangzhou, 510515 China; 4 Guangdong Provincial Key Laboratory of Molecular Oncologic Pathology, Southern Medical University, Guangzhou, 510515 China; 5 Center for Precision Medicine, Guangdong Provincial People’s Hospital, School of Medicine, South China University of Technology, Guangzhou, 510030 China

## Correction to: Protein Cell 10.1007/s13238-021-00821-2

In the original publication Figs. 1, 2 and 3 are incorrectly published, the correct Figs. 1, 2 and 3 are provided in this correction. The original article has been corrected.

**Figure 1:**
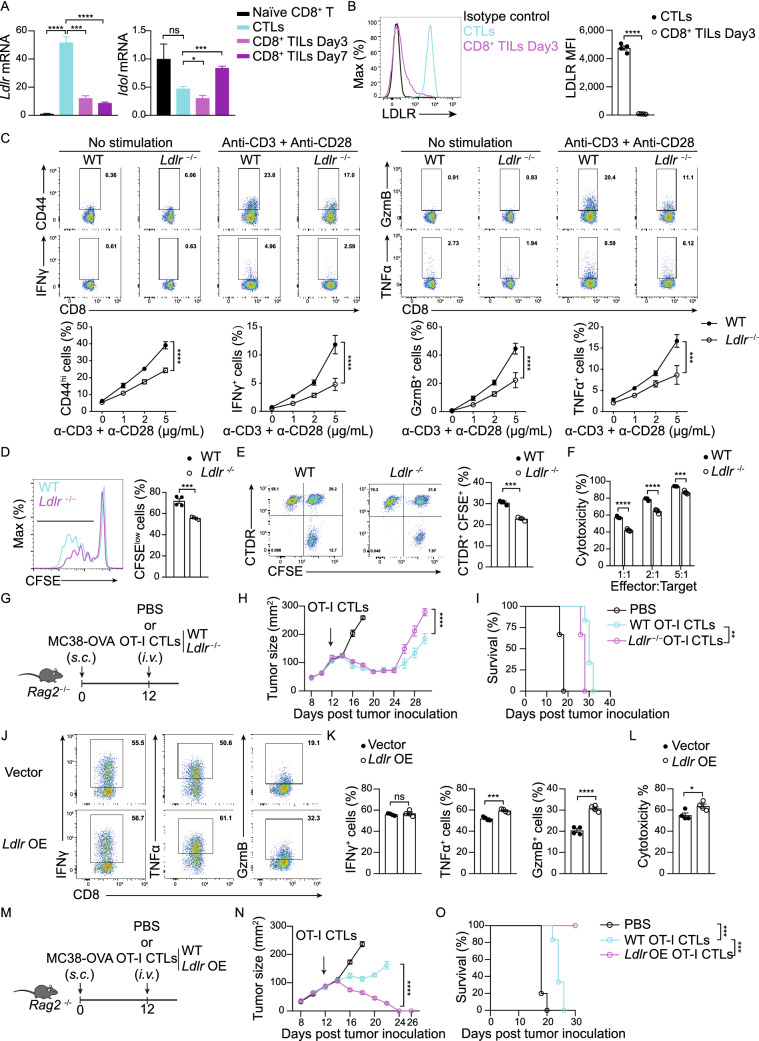
**LDLR deficiency hinders the antitumor activity of CD8**^**+**^ **T cells**. (A) Transcriptional level of genes involved in cholesterol transport in naïve CD8^+^ T cells, CTLs and CD8^+^ TILs (isolated at Day3 or Day7 post CTLs adoptive transfer), (*n* = 4). (B) LDLR expression level on CTLs and CD8^+^ TILs (isolated at Day3 post CTLs adoptive transfer), (*n* = 4). (C) Activation and cytokine/granule productions of WT and *Ldlr*^−/−^ CD8^+^ T cells. Naïve CD8^+^ T cells were isolated from the spleen and stimulated with anti-CD3 and anti-CD28 antibodies for 24 h at indicated concentrations. Data were analyzed by two-way ANOVA (*n* = 4). (D) CD8^+^ T cell proliferation was measured by CFSE dilution assay. CD8^+^ T cells were isolated from the spleen and stimulated with 1μg/mL plate-coated anti-CD3 and anti-CD28 antibodies for 72 h, (*n* = 4). (E) Immunological synapse formation of WT and *Ldlr*^−/−^ CTLs. CFSE-labeled CTLs and CellTracker Deep Red (CTDR)-labeled OVA-pulsed EL4 cells were cocultured for 30 min, (*n* = 3). (F) Cytotoxicity of WT and *Ldlr*^−/−^ CTLs. Splenocytes from WT and *Ldlr*^−/−^ OT-I mice were stimulated with OVA_257–264_ and IL-2 to generate mature CTLs. CTLs were incubated with OVA-pulsed CTDR-labeled EL4 cells and CFSE-labeled non-pulsed EL4 cells for 4 h. The ratio of OVA-pulsed and non-pulsed EL4 cells was calculated to determine the cytotoxicity of CTLs, (*n* = 4). (G) Illustration of adoptive transfer of PBS, WT or *Ldlr*^−/−^ CTLs to MC38-OVA tumor-bearing *Rag2*^−/−^ mice. (H and I) Tumor growth (H) and survival (I) of MC38-OVA tumor-bearing *Rag2*^−/−^ mice with CTL transfer as shown in (G), (*n* = 6). (J and K) Cytokine and granule productions of control and *Ldlr* OE CTLs. *Ldlr* was overexpressed in CTLs with retrovirus infection. The sorted cells were stimulated with 1μg/mL plate-coated anti-CD3 and anti-CD28 antibodies for 4 h, (*n* = 4). (L) Cytotoxicity of control and *Ldlr* OE CTLs. CTLs were incubated with OVA-pulsed EL4 cells and non-pulsed EL4 cells for 4 h, (*n* = 4). (M) Illustration of adoptive transfer of PBS, WT or *Ldlr* OE CTLs to MC38-OVA tumor-bearing *Rag2*^−/−^ mice. (N and O) Tumor growth (N) and survival (O) of MC38-OVA tumor-bearing *Rag2*^−/−^ mice with CTL transfer as shown in (M), (*n* = 5–6). Data were analyzed by *t* test (A, B, D, E, F, K and L) or two-way ANOVA (C, H, I, N and O). *P < 0.05; **P < 0.01; ***P < 0.001; ****P < 0.0001. Error bars denote for the s.e.m

**Figure 2:**
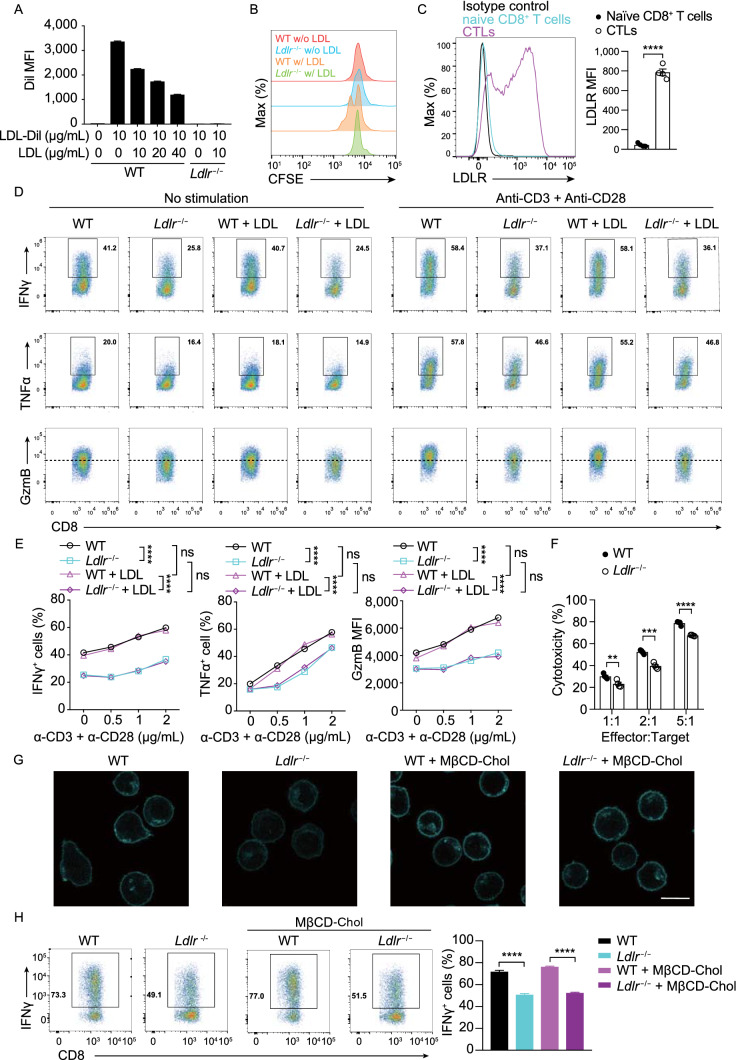
**The regulation of LDLR on CD8**^**+**^ **T cell effector function is not fully dependent on LDL/cholesterol**. (A) LDL uptake of activated WT and *Ldlr*^−/−^ CD8^+^ T cells. CD8^+^ T cells were treated with LDL and LDL-Dil at indicated concentrations. The uptake of LDL-Dil was analyzed by flow cytometry. (B) Proliferation of WT and *Ldlr*^−/−^ CD8^+^ T cells was measured by CFSE dilution. Cells were cultured in medium containing lipoprotein-deficient serum (LPDS) with or without the addition of LDL. (C) LDLR expression of naïve CD8^+^ T cells and CTLs was analyzed by flow cytometry, (*n* = 4). (D and E) Cytokine/granule productions of WT and *Ldlr*^−/−^ CTLs. CTLs were generated from the splenocytes of WT and *Ldlr*^−/−^ mice and pretreated in the medium containing LPDS for 4 h, with or without the presence of LDL. The cells were then stimulated with anti-CD3 and anti-CD28 antibodies for 4 h at indicated concentrations in corresponding medium, (*n* = 4). (F) Cytotoxicity of WT and *Ldlr*^−/−^ CTLs. CTLs were pretreated in the medium containing LPDS for 12 h and cocultured with EL4 cells to determine the cytotoxicity, (*n* = 4). (G) Filipin III staining to analyze cellular cholesterol distribution in untreated or MβCD-coated cholesterol treated WT and *Ldlr*^−/−^ CTLs. Scale bar, 10 μm. (H) IFNγ production of WT and *Ldlr*^−/−^ CTLs. Mature CTLs were generated from the splenocytes of WT and *Ldlr*^−/−^ mice and treated with MβCD-coated cholesterol or not. The cells were then stimulated with 1 μg/mL plate-coated anti-CD3 and anti-CD28 antibodies for 4 h, (*n* = 4). Data were analyzed by *t* test (C, F and H) or two-way ANOVA (E). ns, no significance; **P < 0.01; ***P < 0.001; ****P < 0.0001. Error bars denote for the s.e.m

**Figure 3:**
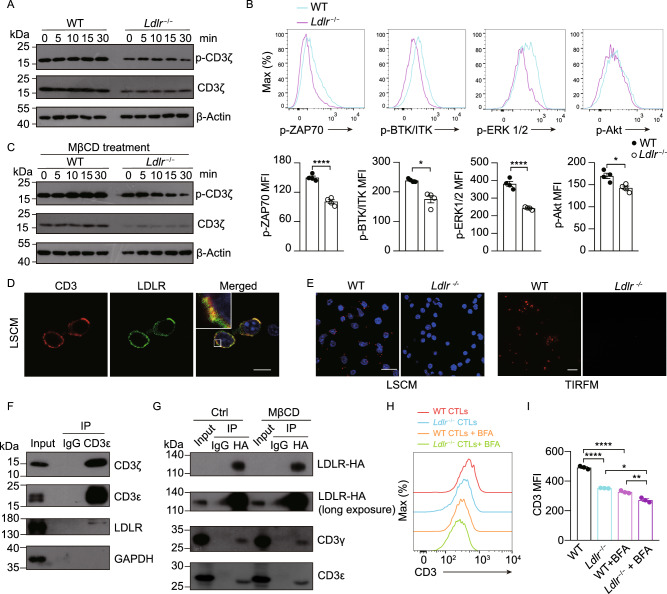
**LDLR interacts with TCR and regulates TCR signaling in CD8**^**+**^ **T cells**. (A) Immunoblotting to detect the phosphorylation of CD3ζ of WT and *Ldlr*^−/−^ CTLs. CTLs were stimulated with 1 μg/mL anti-CD3, anti-CD28, anti-Armenian hamster IgG and anti-Syrian hamster IgG for indicated times. (B) Phosphorylation of ZAP70, BTK/ITK, ERK1/2 and Akt of WT and *Ldlr*^−/−^ CTLs. CTLs were stimulated as in (A) for 10 min. Data were analyzed by *t* test (*n* = 4). (C) Immunoblotting to detect the phosphorylation of CD3ζ of MβCD-treated WT and *Ldlr*^−/−^ CTLs. CTLs were stimulated as in (A). (D) Fluorescence staining of CD3 and LDLR in CTLs. DAPI was shown in blue. Scale bar, 10 μm. LCSM, laser confocal scanning microscopy. (E) Proximity Ligation Assay (PLA) analysis of CD3 and LDLR interaction in WT and *Ldlr*^−/−^ CTLs. Confocal images (left panel, scale bar, 20 μm) and TIRFM images (right panel, scale bar, 10 μm) were shown. Red, PLA signals; Blue, DAPI. TIRFM, total internal reflection fluorescence microscopy. (F) CD3ε was immunoprecipitated (IP) in CTLs and its interaction with LDLR was analyzed by immunoblotting. (G) HA-tagged LDLR was overexpressed in EL4 cells. The EL4 cells were treated with MβCD or not and then HA-tagged LDLR was immunoprecipitated with anti-HA antibody. The interaction between LDLR and CD3 was analyzed by immunoblotting. (H and I) WT and *Ldlr*^−/−^ CTLs were treated with BFA (5 μg/mL) or not for 2 h. CD3 expression was analyzed by flow cytometry. Data were analyzed by *t* test (*n* = 3). *P < 0.05; **P < 0.01; ****P < 0.0001. Error bars denote for the s.e.m

