# Supplementary material for: Potentiating CD8+ T cell antitumor activity by inhibiting PCSK9 to promote LDLR-mediated TCR recycling and signaling
Source: Protein Cell. 2021 Feb 19;12(4):240–60. doi: 10.1007/s13238-021-00821-2 (PMC8018994; doi:10.1007/s13238-021-00821-2)
Supplement: Supplementary file 1 — Supplementary material 1 (PDF 1106 kb) [file 13238_2021_821_MOESM1_ESM.pdf]

Supplementary Figures

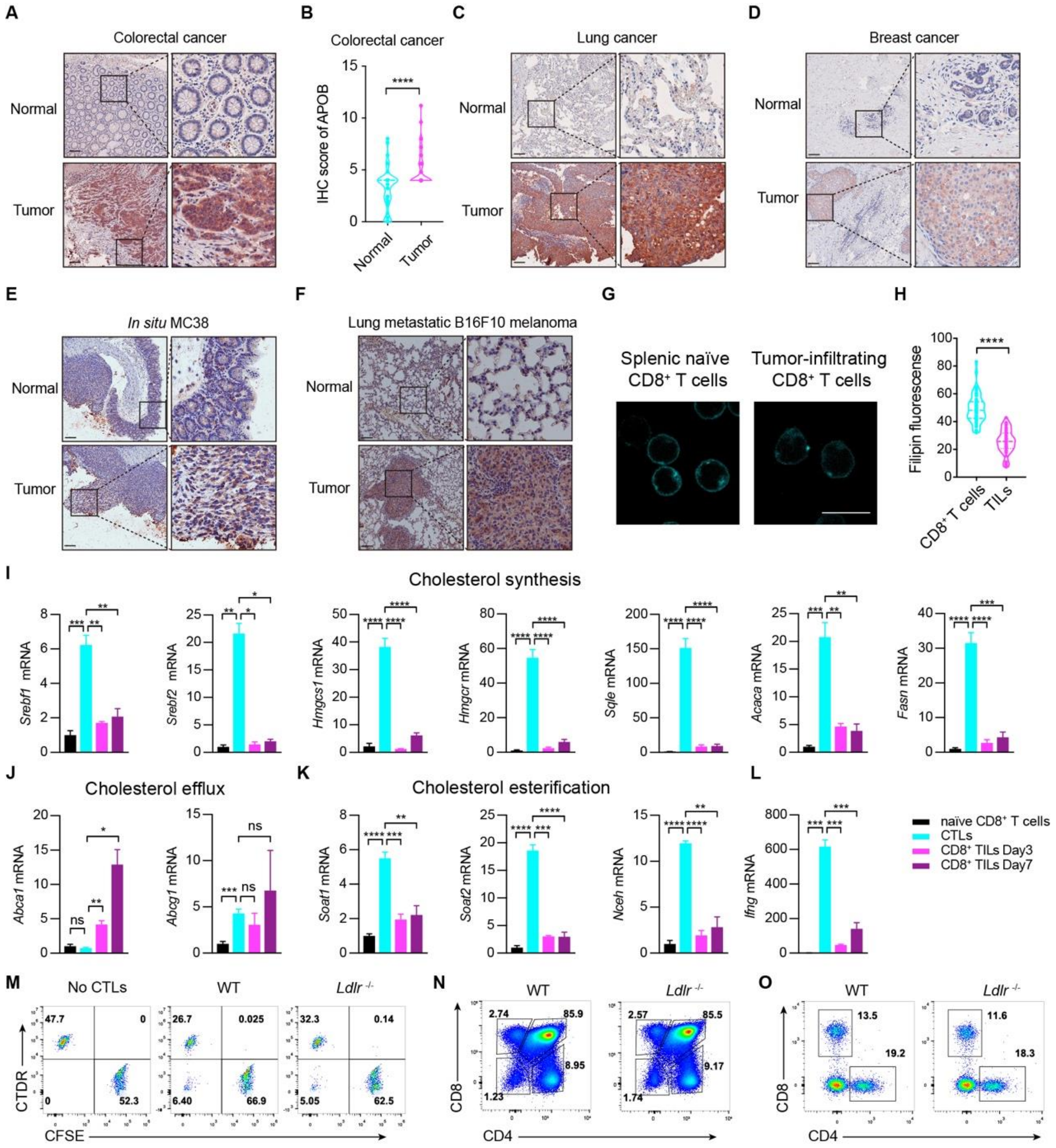

Supplementary figure 1. LDLR deficiency hinders the antitumor activity of CD8$^+$ T cells. (related to Figure 1)

A, B, Human normal colorectal sections or tumor sections were stained with anti-APOB antibody by immunohistochemistry and the abundance of APOB was assessed in (B), (n = 50). **C,** Human normal lung or tumor sections were stained with anti-APOB antibody by immunohistochemistry. **D,** Human normal breast or tumor sections were stained with anti-APOB antibody by immunohistochemistry. **E,** MC38 cells were injected into the cecum of C57BL/6 mice and the tumor sections were stained with anti-APOB antibody by immunohistochemistry. **F,** B16F10 cells were intravenously injected into C57BL/6 mice to induce the lung metastasis of melanoma. The tumor sections were stained with anti-APOB antibody by immunohistochemistry. Scale bar, 120 μm (A-F). **G, H,** Filipin III staining to analyze cellular cholesterol distribution in splenic na ïve or tumor infiltrating CD8$^+$ T cells. The Filipin fluorescence was analyzed in (H). Scale bar, 10 μm. **I-L,** Transcriptional levels of genes involved in cholesterol synthesis (I), efflux (J), esterification (K) and *Ifng* (L) as a control were analyzed by QPCR

in na ïve $CD8^+$ T cells, CTLs and $CD8^+$ TILs (isolated at Day3 or Day7 post CTLs adoptive transfer), (n = 4). **M,** Cytotoxicity of WT and *Ldlr*$^{-/-}$ CTLs. WT and *Ldlr*$^{-/-}$ OT-I CTLs were incubated with OVA-pulsed CTDR-labeled EL4 cells and CFSE-labeled non-pulsed EL4 cells for 4 hours. **N, O,** T cell development analysis of thymic (N) and splenic (O) T cells of WT and *Ldlr*$^{-/-}$ mice. Data were analyzed by *t* test (B, H, I, J, K and L). ns, no significance; *, $P < 0.05$; **, $P < 0.01$; ***, $P < 0.001$; ****, $P < 0.0001$. Error bars denote for the s.e.m.

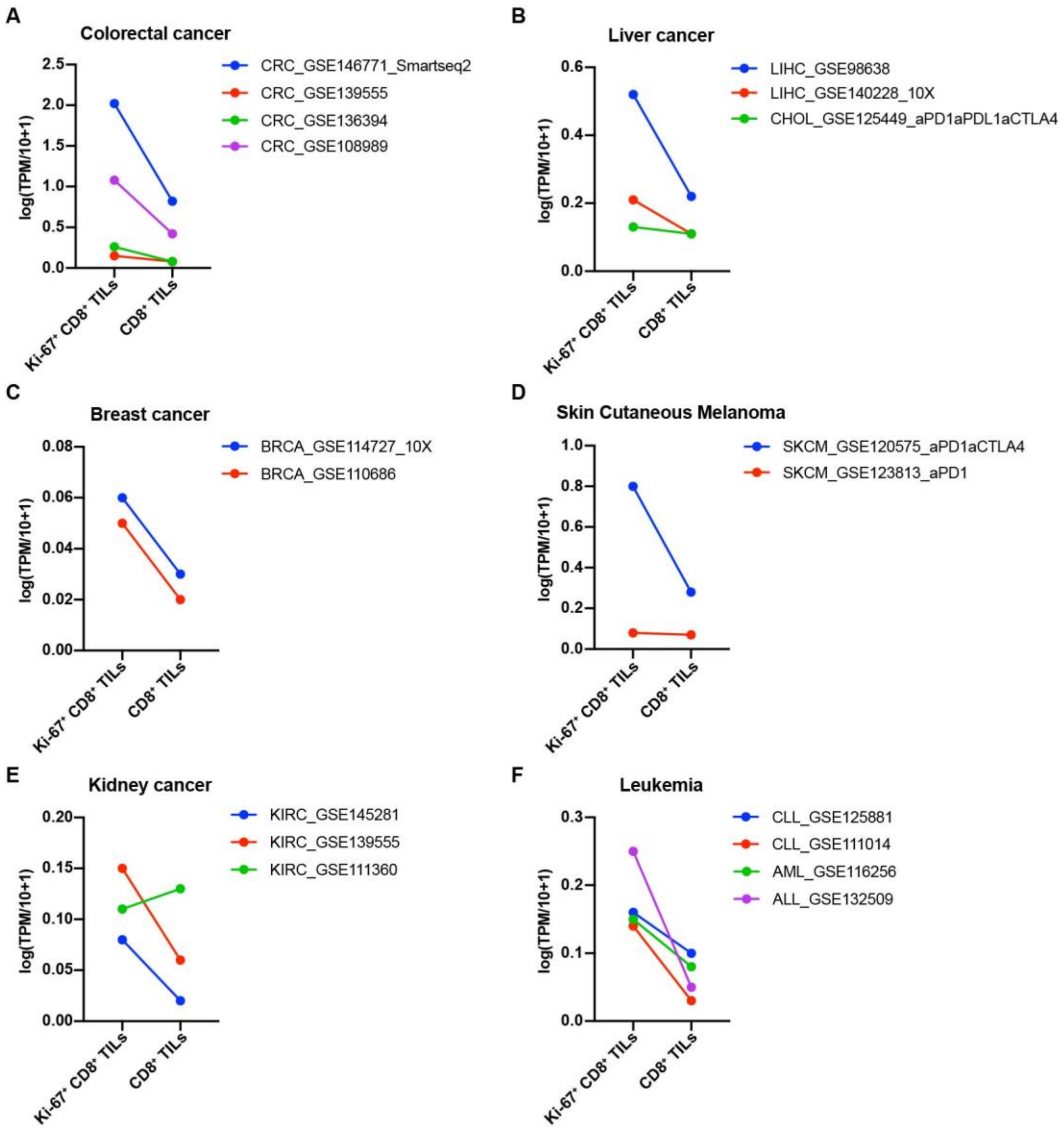

Supplementary figure 2. Active $CD8^+$ TILs showed higher LDLR expression than total $CD8^+$ TILs. (related to Figure 1)

A-F, Transcriptional level of *LDLR* in TILs of cancer patients by using a scRNA-seq database-Tumor Immune Single-cell Hub (TISCH) which contains an atlas of 76 tumor scRNA-seq datasets. $Ki\text{-}67^+$ $CD8^+$ TILs were identified as active $CD8^+$ TILs. $CD8^+$ TILs were identified as total $CD8^+$ TILs.

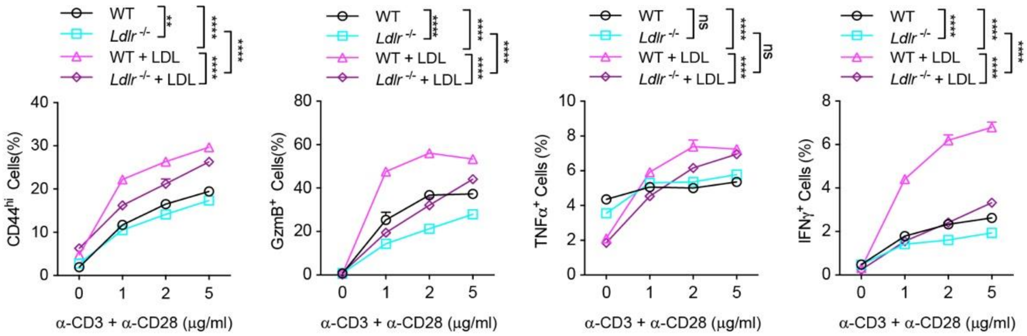

Supplementary figure 3. The regulation of LDLR on CD8$^+$ T cell effector function is not fully dependent on LDL/cholesterol. (related to Figure 2)

Cytokine/granule productions of WT and *Ldlr*$^{-/-}$ CD8$^+$ T cells. Na ïve CD8$^+$ T cells were isolated from the splenocytes of WT and *Ldlr*$^{-/-}$ mice and stimulated with anti-CD3 and anti-CD28 antibodies in the medium containing LPDS for 24 hours, with or without the presence of LDL. Data were analyzed by two-way ANOVA (n = 4). ns, no significance; **, P < 0.01; ****, P <0.0001. Error bars denote for the s.e.m.

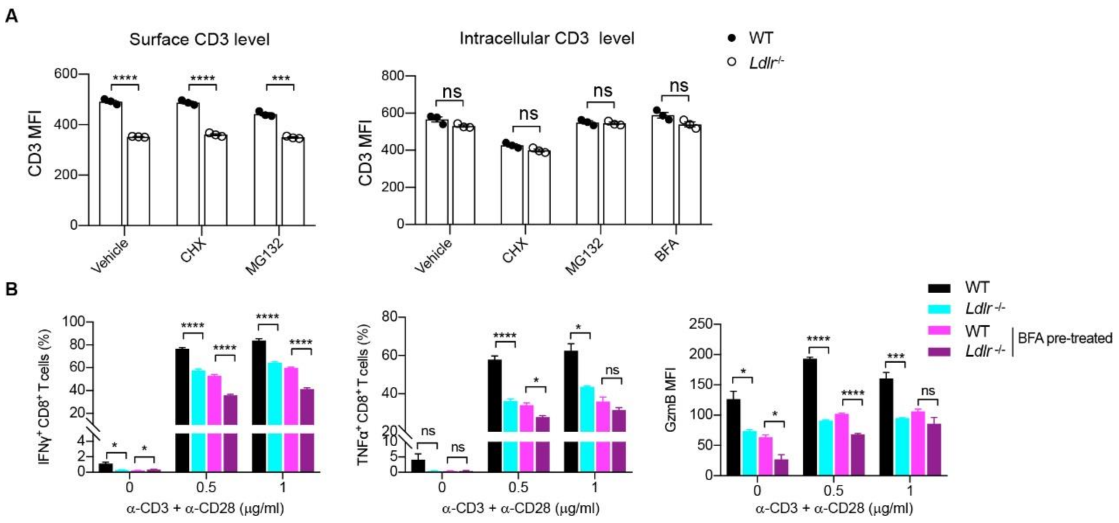

Supplementary figure 4. LDLR interacts with TCR and regulates TCR signaling in CD8$^+$ T cells. (related to Figure 3)

A, CTLs were treated with CHX (50 μg/ml), MG132 (15 μM), BFA (5 μg/ml) or not for 2 hours. Surface level (left panel) and intracellular level (right panel) of CD3 were analyzed by flow cytometry. Data were analyzed by *t* test (n = 3). **B,** Cytokine and granule productions of WT and *Ldlr*$^{-/-}$ CTLs. CTLs were pretreated with BFA (5 μg/ml) or not for 2 hours and stimulated with anti-CD3 and anti-CD28 antibodies for 4 hours at indicated concentrations. Data were analyzed by *t* test (n = 4). ns, no significance; *, $P < 0.05$; ***, $P < 0.001$; ****, $P < 0.0001$. Error bars denote for the s.e.m.

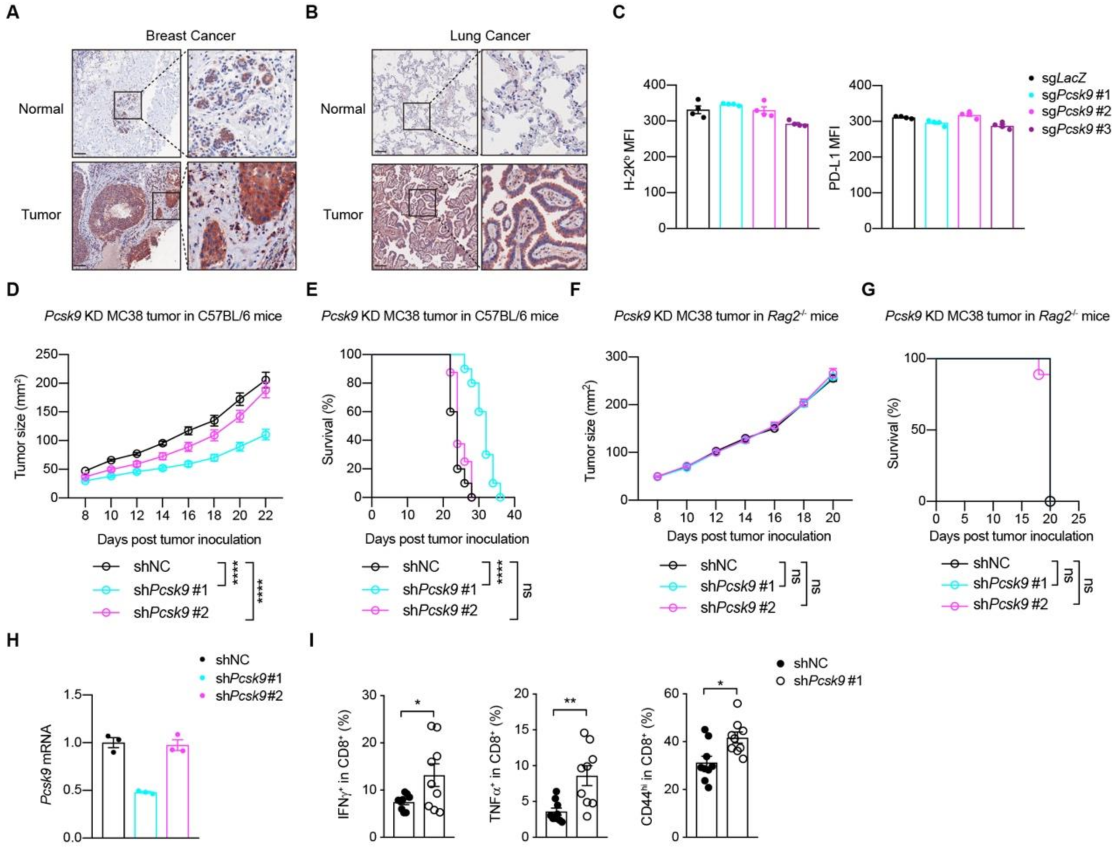

Supplementary figure 5. Tumor-derived PCSK9 inhibits the antitumor activity of CD8^{+} T cells. (related to Figure 4)

A, Human normal breast or tumor sections were stained with anti-PCSK9 antibody by immunohistochemistry. **B,** Human normal lung or tumor sections were stained with anti-PCSK9 antibody by immunohistochemistry. **C,** MHC-I (H-2K^{b}) and PD-L1 expression were analyzed by flow cytometry in *Pcsk9* knockout B16F10 cells. **D, E,** Tumor growth (D) and survival (E) of *Pcsk9* knockdown MC38 tumor-bearing C57BL/6 mice. Data were analyzed by two-way ANOVA (n = 8-10). **F, G,** Tumor growth (F) and survival (G) of *Pcsk9* knockdown MC38 tumor-bearing *Rag2*$^{-/-}$ mice. Data were analyzed by two-way ANOVA (n = 9). **H,** Transcriptional level of *Pcsk9* was measured by QPCR in *Pcsk9* knockdown MC38 cells. **I,** Cytokine productions and activation of tumor infiltrating CD8^{+} T cells isolated from shNC or sh*Pcsk9* MC38 tumors. Data were analyzed by *t* test (n = 9). ns, no significance; *, P < 0.05; **, P < 0.01; ****, P <0.0001. Error bars denote for the s.e.m.

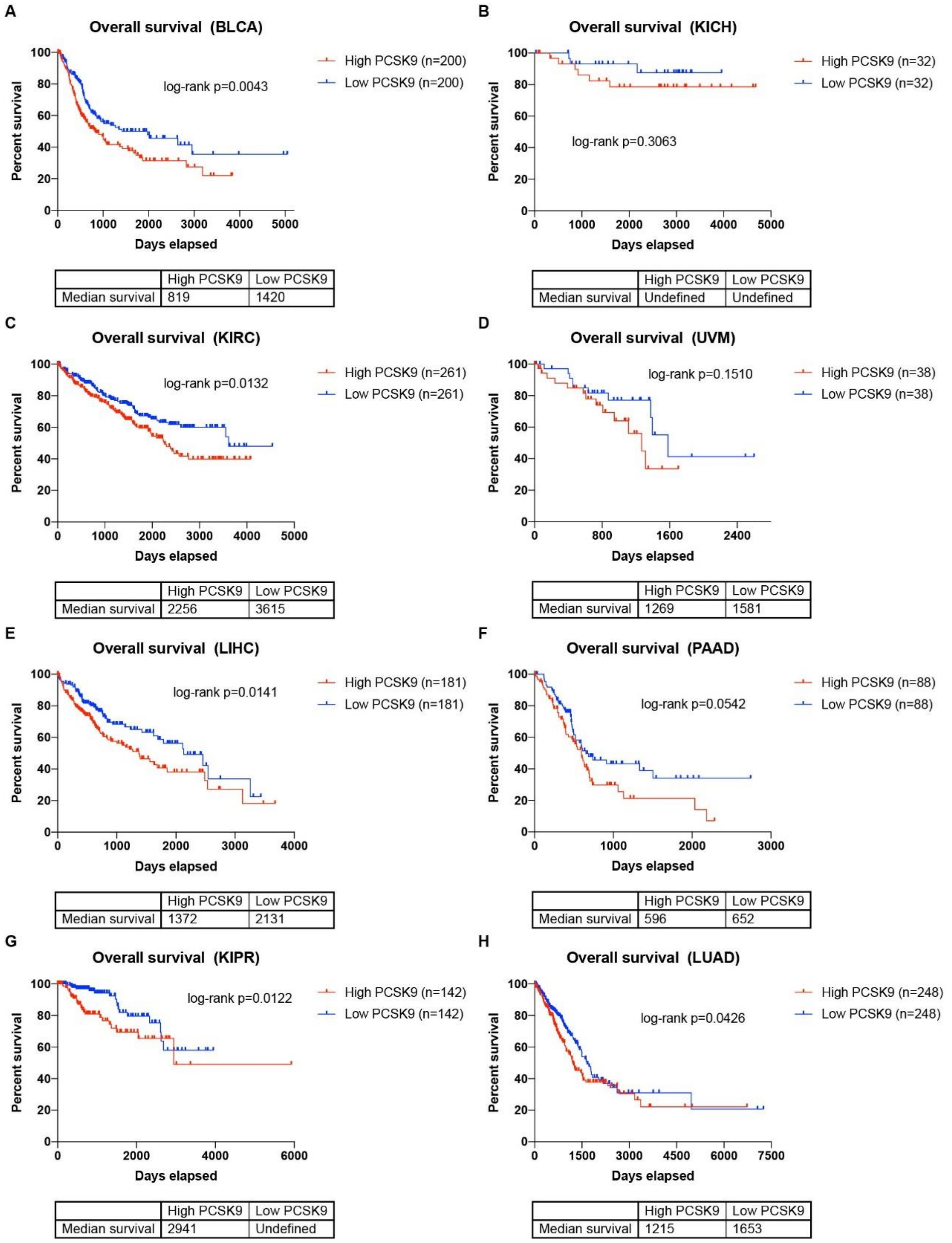

Supplementary figure 6. Prognosis analysis of PCSK9 in clinical data from TCGA database. (related to Figure 4)

The relationship between tumor PCSK9 expression and the survival rate of patients with BLCA (Bladder urothelial carcinoma), KICH (Kidney chromophobe), KIRC (Kidney renal clear cell carcinoma), UVM (Uveal melanoma), LIHC (Liver hepatocellular carcinoma), PAAD (Pancreatic adenocarcinoma), KIPR (Kidney renal papillary cell carcinoma) or LUAD (Lung adenocarcinoma), analyzed in clinical data from TCGA database.

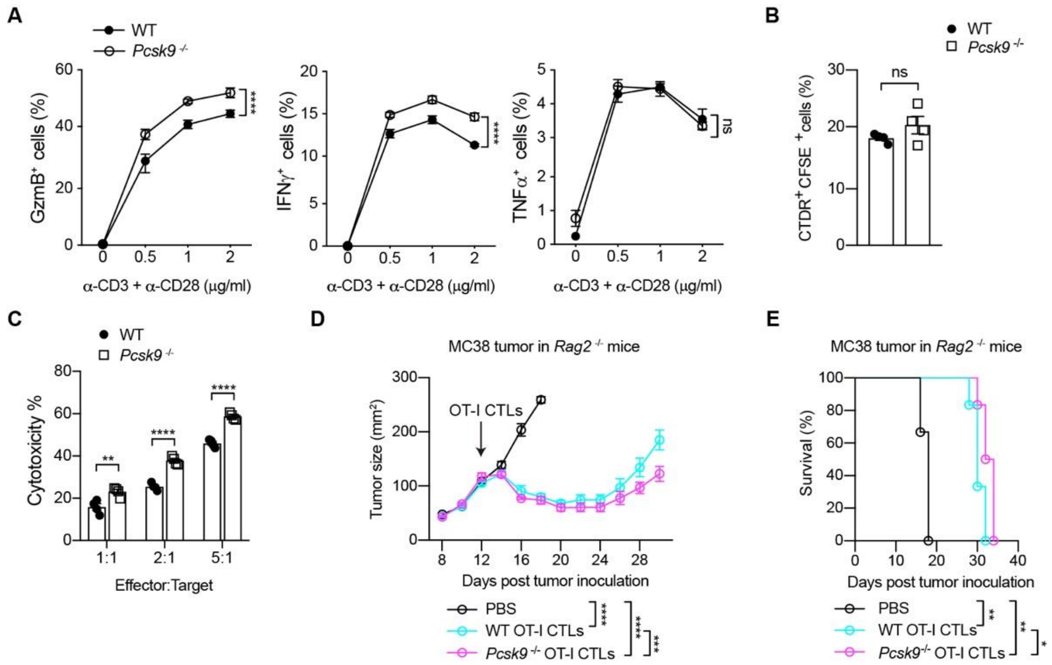

Supplementary figure 7. Inhibiting PCSK9 in CD8$^+$ T cells intrinsically potentiates their antitumor activity. (related to Figure 4)

A, Cytokine/granule productions of WT and *Pcsk9*$^{-/-}$ CD8$^+$ T cells. CD8$^+$ T cells were isolated from the spleen of WT or *Pcsk9*$^{-/-}$ mice and stimulated with anti-CD3 and anti-CD28 antibodies at indicated concentrations for 24 hours. Data were analyzed by two-way ANOVA (n = 4). **B,** Synapse formation of WT and *Pcsk9*$^{-/-}$ CTLs. CFSE-labeled CTLs and CTDR-labeled OVA-pulsed EL4 cells were cocultured for 30 mins. Data were analyzed by *t* test (n = 4). **C,** Cytotoxicity of WT and *Pcsk9*$^{-/-}$ CTLs. CTLs were incubated with OVA-pulsed CTDR-labeled EL4 cells and CFSE-labeled non-pulsed EL4 cells for 4 hours. Data were analyzed by *t* test (n = 4). **D, E,** Tumor growth (D) and survival (E) of MC38-OVA tumor-bearing *Rag2*$^{-/-}$ mice after adoptive transfer of PBS, WT or *Pcsk9*$^{-/-}$ CTLs. Data were analyzed by two-way ANOVA (n = 6). ns, no significance; *, $P < 0.05$; **, $P < 0.01$; ***, $P < 0.001$; ****, $P < 0.0001$. Error bars denote for the s.e.m.

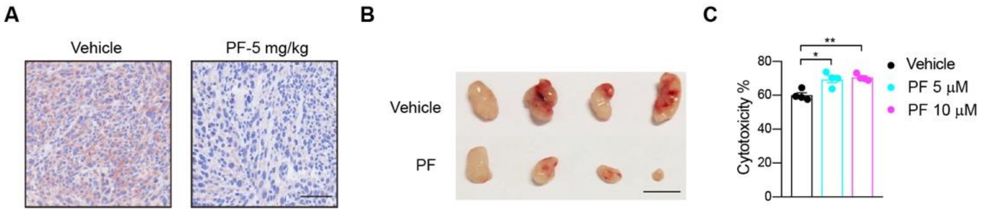

Supplementary figure 8. Inhibiting PCSK9 potentiates the antitumor activity of CD8$^+$ T cells. (related to Figure 6)

A, PCSK9 expression was measured in MC38 tumor sections by immunohistochemistry. MC38 cells were subcutaneously injected into C57BL/6 mice and treated intraperitoneally with 5 mg/kg PCSK9 inhibitor PF-06446846 every 2 days. Scale bar, 50 μm. **B,** MC38 tumors were isolated from PF-06446846 treated C57BL/6 mice and tumor size was shown. Scale bar, 10 mm. **C,** Cytotoxicity of CTLs cocultured with PF-06446846 treated EL4 cells. EL4 cells were pretreated with PF-06446846 for 24 hours and cocultured with CTLs for 12 hours in the presence of PF-06446846. Data were analyzed by *t* test (n = 4). *, $P < 0.05$; **, $P < 0.01$. Error bars denote for the s.e.m.
